# Quantitative high-throughput profiling of snake venom gland transcriptomes and proteomes (*Ovophis okinavensis* and *Protobothrops flavoviridis*)

**DOI:** 10.1186/1471-2164-14-790

**Published:** 2013-11-14

**Authors:** Steven D Aird, Yutaka Watanabe, Alejandro Villar-Briones, Michael C Roy, Kouki Terada, Alexander S Mikheyev

**Affiliations:** 1 Okinawa Institute of Science and Technology, Tancha 1919-1, Onna-son, Kunigami-gun, Okinawa-ken 904-0412, Japan; 2 Okinawa Prefectural Institute of Health and Environment, 2085 Ozato, Ozato Nanjo-shi, Okinawa-ken 901-1202, Japan

**Keywords:** Transcriptome, Illumina, proteome, Mass spectrometry, Venom, Okinawa, Viperidae, Crotalinae, Toxins, Enzymes

## Abstract

**Background:**

Advances in DNA sequencing and proteomics have facilitated quantitative comparisons of snake venom composition. Most studies have employed one approach or the other. Here, both Illumina cDNA sequencing and LC/MS were used to compare the transcriptomes and proteomes of two pit vipers, *Protobothrops flavoviridis* and *Ovophis okinavensis*, which differ greatly in their biology.

**Results:**

Sequencing of venom gland cDNA produced 104,830 transcripts. The *Protobothrops* transcriptome contained transcripts for 103 venom-related proteins, while the *Ovophis* transcriptome contained 95. In both, transcript abundances spanned six orders of magnitude. Mass spectrometry identified peptides from 100% of transcripts that occurred at higher than contaminant (e.g. human keratin) levels, including a number of proteins never before sequenced from snakes. These transcriptomes reveal fundamentally different envenomation strategies. Adult *Protobothrops* venom promotes hemorrhage, hypotension, incoagulable blood, and prey digestion, consistent with mammalian predation. *Ovophis* venom composition is less readily interpreted, owing to insufficient pharmacological data for venom serine and metalloproteases, which comprise more than 97.3% of *Ovophis* transcripts, but only 38.0% of *Protobothrops* transcripts. *Ovophis* venom apparently represents a hybrid strategy optimized for frogs and small mammals.

**Conclusions:**

This study illustrates the power of cDNA sequencing combined with MS profiling. The former quantifies transcript composition, allowing detection of novel proteins, but cannot indicate which proteins are actually secreted, as does MS. We show, for the first time, that transcript and peptide abundances are correlated. This means that MS can be used for quantitative, non-invasive venom profiling, which will be beneficial for studies of endangered species.

## Background

Snakes employ a great variety of biochemical compounds to immobilize, kill, and digest their prey [1,2], although whether venom actually augments assimilation efficiency is a matter of continuing debate [2-6]. Biochemical mechanisms employed in prey envenomation involve a complex interplay between venom chemistry and homeostatic mechanisms in the prey; thus, envenomation success depends upon exploiting the prey’s biochemistry [1]. Venom composition necessarily reflects both the biology of the snake and the nature of its principal prey, factors that change ontogenetically and geographically [7-13].

Biochemical components of a venom participate in one or more of three fundamental envenomation strategies. Two of these are prey immobilization strategies and may be denominated “hypotensive” and “paralytic” strategies [1]. Both serve to limit prey flight, in snake taxa which strike, release, and then track their prey (most viperids), or to overcome prey resistance, in snakes that seize and bulldog their prey (many elapids and all colubrids). The third strategy is digestive and commences degradation of prey tissues internally, even before the prey has been engulfed. Normally, all three strategies operate simultaneously and many individual venom components participate in more than one of them. Each of these three strategies contains interchangeable biochemical constituents. Different venomous taxa employ different combinations of constituents, and no single species employs them all [1].

Snake venom composition can be studied either at the proteomic or the transcriptomic level. Traditionally, snake proteins were sequenced after chromatographic purification, after isolation on polyacrylamide gels, or after cloning cDNA from the venom glands. Although these approaches are typically necessary for studies of protein function, they are laborious, and they are less quantitative than might be desired. Because a relatively small number of individual proteins or clones can be processed at one time, and because techniques vary between labs, comparative analyses of venom chemistry have been difficult [14,15]. Wagstaff et al. [16] found 80% of *Echis ocellatus* venom proteins identified with mass spectrometry in the corresponding transcriptome, but 67% of transcripts were not found in the proteome. In a study of *Bothropoides pauloensis* venom, Rodrigues et al. [17] reported “a low degree of correspondence” between transcriptome and proteome. The degree of correspondence varied, depending upon the protein family. Transcriptome and proteome were in good agreement in regard to bradykinin-potentiating peptides, phospholipases A_2_, and L-amino acid oxidase, but diverged sharply with regard to metalloproteases and C-type lectin-like components. To date, no study has attempted to perform a rigorous statistical comparison of transcriptome and proteome.

Recent technological advances in mass spectrometry and next generation sequencing have greatly simplified both proteomic and transcriptomic studies of snake venoms. Snake venom transcriptomes are now routinely sequenced on a variety of platforms, allowing examination of many more components than has been possible traditionally. In particular, Illumina sequencing, has allowed more accurate quantification of mRNA composition. However, in addition to venom proteins, next generation cDNA sequencing also detects many non-venom components, and erroneous assemblies are another possible source of error. The advent of LC/MS-based venom proteomics permits high throughput screening of venom components [18]. This approach relies on existing databases of protein sequences, and can be limited by the availability of reference data. LC/MS is not typically used to estimate protein abundance. Used together, next generation cDNA sequencing and LC/MS have considerable power, since mass spectrometry can validate cDNA sequencing. However, relatively few venom studies have combined the two tools [14,16,19]. Here both techniques were used to explore the venoms of two Okinawan pit vipers, with the goal of understanding their venom chemistry, and evaluating the performance of LC/MS as a tool for quantifying venom protein composition.

Okinawa, Japan has two native pit vipers, the Okinawa habu (*Protobothrops flavoviridis*) and the himehabu (*Ovophis okinavensis*). Human activities have introduced the Taiwanese habu (*Protobothrops mucrosquamatus*) and the Sakishima habu (*Protobothrops elegans*) as well. The two native species differ in nearly all aspects of their biology. The Okinawa habu is semi-arboreal and can reach lengths of 2.5 m. It is active in the warmer months of the year. In contrast, the himehabu is terrestrial, usually not exceeding 70 cm [20] and is active at temperatures as low as 10°C [20,21].

Most vipers and pit vipers display a well-documented ontogenetic shift from ectothermic prey (primarily lizards and frogs) to endotherms [22-29]. *Protobothrops flavoviridis* follows the usual pattern. Nishimura et al. [30] have documented pronounced ontogenetic dietary shifts in *Protobothrops*. Okinawa habus less than 30 cm in length feed heavily on lizards (36.3% of food items). Amphibians comprise only 3% of the juvenile diet, while house mice (*Mus*) and Horsfield’s shrews (*Crocidura horsfieldi*) constitute another 60.6%. Subadult and adult Okinawa habus (50-90 cm) feed less on amphibians (0.9%) and lizards (2.4%), while birds become an important component (7.9%), and mammals (mice, Horsfield’s and Asian house shrews, and rats) become more important (91.7%). Habus above 1.3 m become exclusive mammal feeders [30].

*Ovophis okinavensis*, by virtue of its small adult size, does not exhibit an apparent dietary shift [20]. While it is technically a dietary generalist [20], its seasonal activity is strongly correlated with frog abundance. In many *Ovophis* populations, frogs comprise nearly 90% of the food items taken by both juveniles and adults [20,21].

The present study employed Illumina cDNA sequencing and LC/MS to investigate the transcriptomes and proteomes of these two native pit vipers. This was done to further illuminate the composition of these two venoms and to ascertain whether the two techniques were congruent.

## Results and discussion

### Transcriptome sequencing, assembly and mapping

After quality filtering, 13,572,340 and 12,184,487 paired-end reads remained, as well as 2,079,603 and 3,110,164 single-end reads, in the *Ovophis* and *Protobothrops* libraries, respectively, which were used for the assembly. When re-mapped to the assembly using RSEM, which used only paired-end read data, 90.4% and 92.0% of the reads were mapped, with 73.4% and 86.7% of these reads being properly paired in alignment. After filtering low-frequency transcripts (less than 1 FPKM), assemblies were reduced from 46,631 and 58,199 transcripts for *Ovophis* and *Protobothrops*, respectively, to 13,998 and 19,970 transcripts.

### Transcriptomes

The *Protobothrops* transcriptome contained partial and complete transcripts for 85 identifiable toxins, representing 21 protein families (Additional file 1: Tables S1 and Additional file 2: Table S4). Expression levels spanned nearly six orders of magnitude. In addition, we identified another 18 sequences in nine more families that are either known to contribute to venom function (e.g. glutaminyl cyclase (QC)), or which potentially do so. The latter group includes tissue enzymes that have been recruited into some venomes (e.g. paraoxonase) and purine and pyrimidine biosynthetic enzymes (e.g. adenylosuccinate synthase) that are extremely important in viperid and elapid venoms, but less so in those of crotalids [31]. While 16 families were represented by a single sequence (Additional file 1: Table S1), others displayed anywhere from 2-21 sequences. Metalloproteases (MPs) (21), serine proteases (SPs) (16), and C-type lectins (CTLs) (12) were the most diversified families in *Protobothrops* venom (Additional file 1: Table S1); however, the most diversified families are not necessarily the most heavily expressed. Forty of the 103 sequences were identical or nearly so, to sequences previously published for this species. Another 51 were most similar to toxins known from other venomous snake taxa. The remaining 12 were most similar to sequences from other vertebrate taxa, including amphibians, lizards, and mammals; thus, in total there were an estimated 63 new sequences for this species, including the 12 that were novel for snakes.

The *Ovophis* transcriptome contained 76 transcripts for identifiable toxins belonging to 19 toxin families (Additional file 3: Tables S2 and Additional file 4: Table S5). Purine and pyrimidine biosynthetic enzymes, acetylcholinesterase, and glutaminyl cyclase comprised another 20 transcripts. The range of expression levels was similar to that seen in the *Protobothrops* transcriptome. In the *Ovophis* transcriptome, SPs were the most diversified family (26 sequences), followed by MPs (10), and CTLs (8). Only one sequence, a phospholipase A_2_, has been previously published for this species. Another 81 sequences were most similar to those of other snakes, while 13 were most similar to sequences from the iguanid lizard, *Anolis carolinensis*. In total, 94 partial and complete sequences reported herein appear to be new for *Ovophis okinavensis*.

**Figure 1:**
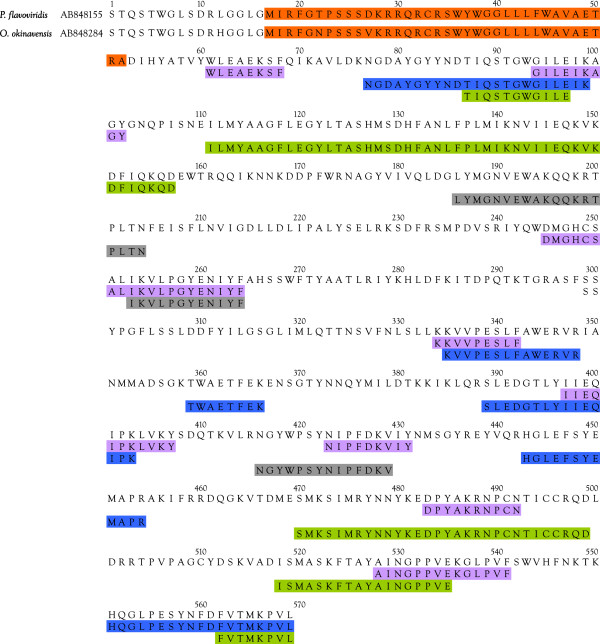
**Abundance of cDNA transcripts in venom glands of *Protobothrops flavoviridis *and *Ovophis okinavensis*, as a percentage of the respective transcriptomes.** Abundant transcripts mask the presence of most venom constituents, some of which are six orders of magnitude (10^6^-fold) less abundant. For the sake of legibility, only toxins comprising ≥1% of either transcriptome are shown here. Toxin class abbreviations are as follows: CRISP, cysteine-rich secretory proteins; CTL, C-type lectin-like proteins; CTL F IX/X, C-type lectin-like activators of Coagulation Factors IX/X; LAO, L-amino acid oxidase; MP, metalloproteases; NGF, nerve growth factor; PLA_2_, phospholipase A_2_; and SP, serine proteases. These two venoms are starkly different in composition. *Protobothrops* venom comprises modest titers of eight toxin families, while *Ovophis* venom consists overwhelmingly of SPs (93.1%) with a lesser quantity of MPs (4.2%). Both venoms contained arrays of lesser constituents, which also varied dramatically between the two species.

The two transcriptomes revealed fundamentally different envenomation strategies (Figure 1; Additional file 5: Table S3). In *Protobothrops* venom glands, phospholipases A_2_ (PLA_2_) (32.1% of all transcripts) and metalloproteases (27.0%) were the dominant constituents, followed by Factor IX/X activators (11.6%), SP transcripts (11.1%), and L-amino acid oxidase (LAO) (9.1%) (Additional file 1: Table S1 and Additional file 5: Table S3; Figure 1). Thus, these five protein classes accounted for 90.9% of all transcripts. In *Ovophis* venom glands, SPs were the dominant component (93.1%), followed by MPs (4.2%), PLA_2_ (0.65%), LAO (0.62%), and C-type lectin-like proteins (CTL) (0.47%), (Additional file 3: Table S2 and Additional file 5: Table S3; Figure 1). Thus, in *Ovophis*, the dominant five classes comprise 99.0% of total venom transcripts.

Significant differences are also evident in terms of minor components (Additional file 5: Tables S3, Additional file 2: Table S4 and Additional file 4: Table S5). Three-finger toxins (3FTx), paraoxonase, vespryn, and waprin transcripts were found in the *Protobothrops* transcriptome, but were absent in *Ovophis*. Five acetylcholinesterase (AChE) transcripts and crotasin-like transcripts were found in the *Ovophis* transcriptome, but not in that of *Protobothrops*. Glutaminyl cyclase (QC) cDNA was detected at lower levels in the *Ovophis* transcriptome. 5’-nucleotidase, CRISP, CTL, nerve growth factor (NGF), and phosphodiesterase (PDE) transcripts were significantly more abundant in *Protobothrops* venom, whereas dipeptidyl peptidase IV (DPP IV) was more abundant in *Ovophis* venom glands (Additional file 5: Table S3).

Both transcriptomes also contained numerous transcripts that appear unrelated to envenomation (Additional file 6: Table S6 and Additional file 7: Table S7). The majority of these appear to be cellular proteins and were transcribed at very low levels. Peptides were also isolated for many of these (Additional file 6: Table S6 and Additional file 7: Table S7). Whether such constituents make a significant contribution to envenomation is unknown, but it seems unlikely.

### Proteomes

Peptides were isolated from 100% of venom or venom-related transcripts that were more abundant than contaminants (e.g. human keratin) (Additional file 1: Table S1 and Additional file 3: Table S2). Peptides were also isolated from at least 18 transcripts in the two transcriptomes that occurred below contaminant levels (Additional file 2: Table S4 and Additional file 4: Table S5).

### Comparison between proteomic and transcriptomic data sets

**Figure 2:**
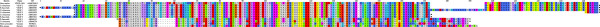
**Gene expression in the venom glands correlates well with protein abundance in the venom.** In both cases the correlation was strongly significant, although roughly half of the variance remained unexplained. These data show that mass spectrometry can provide quantitative data on protein abundance in snake venom proteomes. A similar pattern can be seen using publicly available snake venom proteins from NCBI as a protein reference (Additional file 8: Figure S1), suggesting that this technique should also work without species-specific transcriptomic data.

Although one would expect to find strong correlations between venom gland mRNA and protein profiles, such a link has been elusive [32]. Lack of correlation between the two types of data may be due to biological reasons, such as biased processing of messenger transcripts. Alternatively, purely technical reasons may have prevented accurate estimation of cDNA or protein abundance, particularly in early studies in which sequencing by the Sanger method limited the number of clones. Although our measure of protein abundance was relatively crude, we were nonetheless able to detect a correlation (*Protobothrops flavoviridis* r = 0.77, p < 2.2e^-16^; *Ovophis okinavensis* r = 0.70, p < 1.1e^-12^) between mRNA and venom protein levels (Figure 2).

We were able to confirm the correlation between proteomic and transcriptomic estimates of protein abundance using publicly available data from NCBI (Additional file 8: Figure S1). There were no proteins detected in the NCBI data set that were missing from our transcriptome, suggesting that we were able to capture all of the transcriptional diversity. The robustness of the result also argues against a spurious correlation driven by poor assembly and mapping of low FPKM transcripts.

The correlation, although significant, explained only about half of the variance in the data. Apparent differences between mRNA and protein levels may stem from several factors, both biological and analytical. For example, although tissue and venom samples were taken from the same individuals, they were taken at different times. If venom components are synthesized at different rates the two measurements might not agree. Likewise, it is possible that due to extensive post-translational modification of many venom components, not all messenger transcripts have an equal chance of becoming mature proteins. It is also likely that our measure of protein abundance is not sufficiently precise, due perhaps to biased cleavage of proteins or biases in ion detection during LC/MS. Proteins differ in their susceptibility to enzymatic digestion. Even though three proteases (chymotrypsin, Glu-C, and trypsin) were used, few proteins were digested equally well by all three. More abundant peptides are much more likely to be detected by mass spectrometry than others. Lastly, it is probable that incomplete transcripts stemming from the short read length diminished the strength of the correlations. Newer Illumina sequencer models (e.g., MiSeq) now boast read lengths as great as 500 bp, which may mitigate this problem in future studies.

There are a large number of toxin and potential toxin transcripts that are expressed at near-zero levels (Additional file 1: Table S1, Additional file 2: Table S4, Additional file 3: Table S2, Additional file 4: S5, Additional file 5: Table S3). These include 3-finger toxins, AChE, acid phosphomonoesterase, crotasin-like proteins, paraoxonase, tissue factor pathway inhibitor (TFPI), vespryns, waprins, and many MP and SP transcripts. There is no evidence that many of these are actually translated, or, if they are, they are not a substantial proportion of the proteome. This raises the question of what function these transcripts may now have, or may have had previously. Are these merely tissue transcripts that have not actually been incorporated into the venome? How high an expression level would be required before novel venom proteins would have selective value, or would be under selective pressure? Undoubtedly selective pressure would vary with the biochemical envenomation strategy employed by the taxon in question, and also upon the nature of the contribution made by a given toxin to that strategy. Given the massive overkill that most venoms generate, it is likely that a substantial contribution would be required to generate much selective pressure. It also seems likely that there would be more selective pressure to increase prey immobilization efficiency than acute toxicity or assimilation efficiency.

### Major venom constituents

#### ***Metalloproteases***

Snake venom MPs are presently classified into four groups, according to domain structure and size: P-I MPs possess a metalloprotease domain only and are largely hemorrhagic; P-II MPs are larger, with metalloprotease and disintegrin domains; P-III enzymes have metalloprotease, disintegrin, and cysteine-rich domains; and P-IV enzymes have a lectin-like domain linked by disulfide bonds to a P-III structure [33]. The structural complexity of P-III enzymes has resulted in greater functional diversity. They promote hemorrhage, inflammation, apoptosis, and prothrombin activation, while inhibiting platelet aggregation. As a general rule, P-III enzymes are more potent hemorrhagins than P-I enzymes [33]. In addition to degrading vascular endothelial basement membrane (hemorrhagins), collectively, MPs exhibit diverse and variable combinations of activities. Some anticoagulant metalloproteases degrade only the fibrinogen Aα chain [34], while others degrade one or more chains of both fibrinogen and fibrin with varying specificity [34-36]. Still others release histamine [37], antagonize platelet aggregation by different mechanisms [38-41], or activate [42] or digest plasminogen [43]. Some are procoagulant, possessing Factor Xa-like activity [44]. Few laboratories have exhaustively assayed MPs for potential biological and biochemical activities; thus, inferring such functions from structure is almost impossible. The same may be said of SPs.

The *Protobothrops* transcriptome contained transcripts for twelve P-II MPs and nine P-III MPs. One of the P-II enzymes (MP 01) constituted 11.06% of all toxin transcripts and collectively P-II transcripts accounted for barely ~11.1% of the transcriptome (Additional file 9: Figure S2; Additional file 1: Tables S1, Additional file 5: Table S3, and Additional file 2: Table S4). P-III transcripts were more abundant, comprising 15.8% of all transcripts. Three sequences were homologous to hemorrhagic proteases HR1A and B [45,46]. The *Ovophis* transcriptome included seven P-II transcripts and three P-III transcripts. In *Ovophis*, P-II transcripts represented only 1.6% of all transcripts (Additional file 3: Table S2 and Additional file 4: Table S5). P-III transcripts added another 2.6%. Thus MPs comprised a mere 4.2% of the *Ovophis* transcriptome, compared to 26.9% in *Protobothrops* (Figure 1, Additional file 9: Figure S2 and Additional file 10: Figure S3; Additional file 3: Table S2, Additional file 5: Table S3, and Additional file 4: Table S5).

Of the 21 *Protobothrops* MPs, peptides were sequenced by mass spectrometry for 15, with coverage ranging from 31.1-91.4% of the respective transcripts (Additional file 1: Table S1). Peptide coverage of *Ovophis* MPs ranged from 26.9-80.6% (Additional file 3: Table S2).

#### ***Phospholipases A***_***2***_

The *Protobothrops* transcriptome contained four transcripts for PLA_2_s (Additional file 1: Table S1), including a Lys-49 myotoxin (PLA_2_ 4) [Accession #: AB851948] and a weak neurotoxin similar to trimucrotoxin [47] (PLA_2_ 2) [AB848131]. PLA_2_ 1 [AB848154] accounted for 26.7% of all transcripts, while PLA_2_ 2 amounted to an additional 5.5% (Additional file 1: Table S1 and Additional file 2: Table S4). The *Ovophis* transcriptome contained two PLA_2_ transcripts; however, the more abundant transcript, PLA_2_ 1, comprised only 0.65% of the transcriptome (Additional file 3: Table S2 and Additional file 4: Table S5) [AB848270]. Peptides sequenced by mass spectrometry covered 98.3% of PLA_2_ 1, but no peptides were found for the minor transcript.

#### ***Serine proteases***

Of the 18 SP transcripts in the *Protobothrops* library (Additional file 1: Table S1 and Additional file 2: Table S4), only two (SP01 and SP 12) (FPKM% 2.6 and almost zero, respectively) can be confirmed as complete (Additional file 11: Figure S4). Several transcripts appear to encode dysfunctional SPs. For instance, SP16 encodes 36 residues and is bracketed on both ends by stop codons (Additional file 1: Table S1; Additional file 11: Figure S4). Given that it was expressed at a very low level and that no peptides were sequenced by mass spectrometry, we think it is unlikely to play any role in envenomation.

SP01, the most abundant SP transcript, corresponds to a protein that appears in the literature under the names of habutobin [48-51] and flavoxobin [52-56], a weakly thrombin-like enzyme of 242 amino acids that specifically releases fibrinopeptide A from fibrinogen [55]. No information is available with regard to possible kallikrein-like activity. However, Yamamoto et al. [56] found that flavoxobin is an active C3-convertase that selectively releases C3b and C3a. It remains active in blood containing endogenous protease inhibitors, and promotes massive C3 consumption, and to a lesser extent, C5 cleavage. A kinin-releasing enzyme, flavoviridiobin, is also known from this venom [57]; however, since no sequence data are available, we cannot identify it among our transcripts. Enzymatic digests of crude venom effected with trypsin, chymotrypsin, and Glu-C (Pierce) yielded peptides that accounted for 94.6% of the primary structure of SP01 (Additional file 1: Table S1; Additional file 11: Figure S4). Reasonable peptide coverage of transcripts as minor as 0.24% (SP11, 50.0%) was achieved (Additional file 1: Table S1).

In contrast to the *Protobothrops* library, the *Ovophis* library contained transcripts for 26 different SPs (Additional file 1: Table S1 and Additional file 2: Table S4; Additional file 12: Figure S5). Peptide coverage of 36% or above was achieved for 22 of these, with coverage above 70% for 11 of them. Two transcripts (SP09 and 10) appear to be plasminogen activators, while SP20 is most similar to a kinin-releasing enzyme from the venom of *Bothrops jararaca*. Serine proteases display numerous amino acid substitutions, and the structural determinants that specifically account for kinin-releasing activity are unknown [58]. The difficulty in assigning pharmacological activities to specific sequence variations is immediately apparent upon a cursory examination of Additional file 11: Figure S4 and Additional file 12: Figure S5.

Wu et al. [59] reported a novel class of inactive serine protease homologs (SPH) that displayed an arginine substitution for His-43 of the catalytic triad. SP13 was the only serine protease in our *Protobothrops* library that showed this His → Arg mutation (Additional file 12: Figure S5, position 126); however, the *Ovophis* library contained eight transcripts with His → X substitutions (Additional file 12: Figure S5, position 101). Two of these, SP08 and SP22 showed His → Lys substitutions; two putative thrombin-like enzymes, SP16 and SP17 displayed His → Asn substitutions, and SP07 had a His → Ala substitution. Numerous other sequence differences appear in that transcript as well (Additional file 12: Figure S5). SPHs from other sources have been shown to possess diverse activities, so it is possible that inactive SPs in venoms have developed other unknown functions, some of which may be specialized for particular prey types.

An inactive catalytic triad is but one of many structural differences manifested by *Ovophis* SPHs (Additional file 12: Figure S5). Almost all of the cysteine residues are in different positions as well (Cys-102 has only moved to position 100, but most have shifted substantially more), although within the group, most residues are conserved across most sequences. SP07 is a marked exception in the latter regard (Additional file 12: Figure S5). Another oddity among these sequences is that four of them (SP01, 07, 23, and 26) are truncated C-terminally with stop codons, despite the fact that SP01 and 07 display expression levels of 9.6 and 7.1%, respectively. Wang et al. [60] reported that a Kentucky population of *Crotalus horridus* lacks an acidic PLA_2_ because the codon for Tyr^22^ has mutated into a stop codon. They concluded that low PLA_2_ expression levels in most *Crotalus horridus* venoms can be attributed to translation blockage. At this point, it is difficult to know how widespread this phenomenon might be, but it is apparent that these two *Ovophis* SPs are translated effectively since they had ample peptide coverage (Additional file 3: Table S2).

#### ***L-amino acid oxidase***

The *Protobothrops* transcriptome included two transcripts for L-amino acid oxidase [AB848142, AB848143], comprising 2.3% and 6.8% of all transcripts, respectively (Figure 1; Additional file 1: Table S1). A single LAO transcript was present in *Ovophis* glands [AB848269], representing 0.6% of the transcriptome (Additional file 3: Table S2). Peptides accounting for 84.6% and 70.8% of *Protobothrops* LAO 1 and LAO 2, respectively, and 78.7% of the *Ovophis* LAO transcript sequence was identified by mass spectrometry (Additional file 1: Table S1 and Additional file 3: Table S2).

### Minor venom constituents

#### ***Cysteine-rich secretory proteins***

Two CRISPs were identified in the *Protobothrops* transcriptome (Additional file 1: Table S1 and Additional file 2: Table S4). CRISP 1 [AB848115], (FPKM = 3.9%) for which a complete transcript was obtained, is identical to triflin [61], but CRISP 2 [AB851959] aligns best with a CRISP bearing an EGF-like calcium-binding domain from the venom of *Crotalus adamanteus*[62] (Additional file 2: Table S4). However, the putative 39-residue EGF domain in the *C. adamanteus* toxin does not align well with the corresponding region of the *Protobothrops* transcript. The latter contains only four acidic residues, compared with nine in the *C. adamanteus* sequence. Only three of the five *C. adamanteus* cysteine residues match, and the two sequences require a two-residue gap to achieve even this poor alignment. Therefore, we think it unlikely that there is a functional EGF-like calcium binding domain in the *Protobothrops* toxin. Moreover, no peptides were sequenced for this odd CRISP, whereas 84.6% of CRISP 1 was sequenced.

A single, complete CRISP transcript (FPKM = 0.2%) was identified in the *Ovophis* transcriptome (Additional file 2: Table S2) [AB848276], but sequenced peptides accounted for 89.0% of its primary structure. It was most similar to a CRISP from the venom of *Bothriechis schlegelii* [GenBank: ACE73559.1]*.*

CRISPs are generally not abundant components of snake venoms, but they are widely distributed taxonomically. Ablomin (*Gloydius blomhoffii*), triflin (*Protobothrops flavoviridis*) and latisemin (*Laticauda semifasciata*) are L-type Ca^2+^ channel antagonists of depolarization-induced arterial smooth muscle contraction, but they do not affect caffeine-induced contraction [61]; thus they promote vasodilation and hypotension. Tigrin from “venom” of the Japanese colubrid, *Rhabdophis tigrinus*, affected neither. This is probably because *Rhabdophis* venom glands are not secretory in nature. Instead, *Rhabdophis* glands sequester toxins from the blood stream that are derived from the toads that *Rhabdophis* eats [63]. Thus, tigrin is most likely an amphibian toxin, intended for oral or gastric activity, and not a snake toxin, designed for direct vascular action. In contrast, patagonin, a CRISP isolated from the venom of the colubrid, *Philodryas patagoniensis*, damaged murine skeletal muscle [64].

#### ***Nerve growth factor***

Both habu transcriptomes contained a single, complete transcript for nerve growth factor [Pf: AB848144; Oo: AB848271] (Additional file 1: Table S1 and Additional file 3: Table S2). The *Protobothrops* transcript accounted for 0.7% of all transcripts while the *Ovophis* transcript accounted for 0.5%. Both transcripts are translated and peptides were isolated by mass spectrometry. NGFs function as arginine esterases [65,66], so they probably contribute to venom hypotensive activity via nitric oxide liberation and histamine release [67,68]. Mouse salivary NGFs activate plasminogen, their only known action upon a biologically important, non-neural substrate [69,70], but it is not clear whether snake venom NGFs can also do this. If so, they would hinder blood clotting.

#### ***C-type lectins***

Snake venom C-type lectins, or snaclecs [71] are commonly found in pit viper venoms. These proteins differ from classical C-type lectins in that they lack the calcium and sugar-binding loop and instead bind to a large variety of proteins and receptors involved in hemostasis, including coagulation factors IX and X and various blood platelet receptors [72]. They may consist of one, two, or four αβ heterodimers, and in some cases, the heterodimer is incorporated into a metalloprotease [73]. In many CTLs, dimers are formed by domain swapping between subunits [73].

CTL pharmacology is quite complex. Taniuchi et al. [74] found that flavocetin A actually induces formation of small platelet aggregates, but the dose-dependency is bell-shaped, with a maximum effectiveness at 1-2 μg/mL. Clemetson [72] lamented that because so much venom research is now done at the transcriptional level, the protein chemistry and pharmacology necessary to understand CTL diversity has lagged way behind. In reality, the same could also be said of any other toxin family that shows significant diversification, such as 3FTxs, SPs, MPs, and PLA_2_s.

Venom C-type lectins may activate platelets or inhibit platelet activation, but either mechanism serves the function of inducing thrombocytopenia. Because C-type lectins are non-enzymatic, a 1:1 stoichiometry exists between these toxins and their targets. Clemetson [72] noted that for this reason, it is much more efficient to clear platelets by activating them than by inhibiting them. However, different species of snakes employ both strategies, and it is probably necessary to look at all the toxins in a given venom that impact hemostasis, before drawing any conclusions.

Twelve *Protobothrops* CTL transcripts included three α-chains and three β-chains homologous to flavocetin A, an (αβ)_4_ inhibitor of von Willibrand factor-induced, GP1B-mediated platelet aggregation [75,76] and convulxin, a potent (αβ)_4_ inducer of platelet aggregation that binds to GPVI [73] (Additional file 13: Figure S6; Additional file 1: Table S1 and Additional file 2: Table S4). One of the flavocetin A-like α-chains (CTL03α) and CTL07 F IX/X displayed a number of sequence differences, including an unusual C-terminus (CKFLRPR). Whether these have any pharmacological significance is unknown. In addition to toxins that target blood platelets, there were five A chains and one B chain for proteins that bind to coagulation Factors IX/X (Additional file 1: Table S1 and Additional file 2: Table S4). Factor IX/X binding proteins inhibit blood coagulation by blocking the host clotting cascade [77].

Seven *Ovophis* CTL transcripts apparently all encode proteins that affect platelet activation (Additional file 3: Table S2 and Additional file 4: Table S5; Additional file 13: Figure S6). They are homologous to flavocetin A and convulxin. We did not discover any *Ovophis* transcripts that encode anticoagulant Factor IX/X-binding proteins. Our *Ovophis* cDNA library contained one α-chain, CTL1α, similar to the α-chain of flavocetin A (*Protobothrops flavoviridis*) and the convulxin A- and C-chains (*Crotalus durissus terrificus*) (Additional file 13: Figure S6). CTL1α is most like crotacetin (*Crotalus durissus terrificus*. It represented 0.16% of all transcripts. In addition, there were six β-chains, homologous to the flavocetin A β-chain and the convulxin B- and D-chains (Additional file 13: Figure S6; Additional file 3: Table S2). Together these seven CTLs represented 0.47% of all transcripts.

#### ***Bradykinin-potentiating peptides***

**Figure 3:**
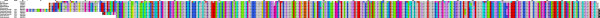
**Alignment of the *Protobothrops flavoviridis *[AB851926] and *Ovophis okinavensis *[AB852004] BPP/CNP transcripts with those of Higuchi et al. **[78]**for *Protobothrops flavoviridis *and *Gloydius blomhoffii. ***Peptides posited by Higuchi et al., or actually sequenced by mass spectrometry in the present study are shown in red. Possible additional BPPs identified in the present study are shown in violet. CNP sequences are highlighted in aqua.

A single bradykinin-potentiating peptide (BPP) was sequenced from *Protobothrops* venom using mass spectrometry (QSKPGRSPPISP) (Additional file 1: Table S1), confirming the existence of a BPP proposed by Higuchi et al. [78], on the basis of a cDNA transcript. A second possible BPP (VVVQPHESPAGGTTA) was also sequenced, but to date, no other BPPs have been found with proline immediately after the N-terminal pyroglutamic acid, making this sequence suspect. Moreover, the VVV-sequence, N-terminal to the glutamine, and the C-terminal AGGTTA-sequence are highly questionable. Possibly this peptide could be processed to QPHESP. This possible BPP is located at the C-terminus of our BPP transcript; however, our BPP transcript is incomplete, since it lacks a stop codon and it does not include the C-type natriuretic peptide-coding region reported by Higuchi et al. [78] (Figure 3).

Our *Protobothrops* transcript [AB851926] also contains the second BPP sequence reported by Higuchi et al. [78], although this BPP was not identified by mass spectrometry. They posited the existence of two BPPs based on the assumptions that such sequences should possess glutamine at the N-terminus and proline at the C-terminus, and should be about 11 residues in length. In fact, BPPs from three to 14 residues have been reported [79,80] (Additional file 14: Figure S7). Both the Higuchi *Protobothrops* transcript and ours suggest another probable BPP with the sequence QWMPGGRPPHHIPP (Figure 3). The *Gloydius* transcript of Higuchi et al. [78] also contains a tripeptide (QWS) that occurs in five locations at the end of the BPPs that they predicted (Figure 3). Two tripeptides from *Bothrops insularis* venom having pyroglutamic acid at the N-terminus (QQK, QKW) were sequenced by Cintra et al. [79], and these peptides were shown to have bradykinin-potentiating activity on guinea pig ileum (Additional file 14: Figure S7). It is possible that the peptide QWS is likewise biologically active. Other tripeptides are found in the Higuchi *Protobothrops* and *Gloydius* transcripts and in our *Ovophis* transcript. These have the sequences QER (*Protobothrops*) and QAR (*Gloydius* and *Ovophis*) (Figure 3, residues 281-283). All of these are immediately N-terminal to nonapeptides that could also be BPPs (Figure 3, residues 284-292). These sequences are as follows: Pf, QKWGRMVQP; Gb, QNWARMVNP; Oo, QKWGRMVPP.

In addition to being truncated on the C-terminal end relative to the Higuchi transcript, our transcript displays a significant N-terminal extension, containing three additional possible BPPs (Figure 3). These have the sequences QRRVHGGERIWP, QSARLDSTRLGSAP, SRPPSLPAPAQP; however, additional work will be necessary to determine whether these sequences are actually hypotensive and whether they are actually expressed in habu venom.

Our *Ovophis* BPP transcript [AB852004] displayed a C-terminal stop codon, but was incomplete on the N-terminal end. However, the *Ovophis* transcript did contain a sequence for a C-type natriuretic peptide (GVAKGCFGLKLDRIGTMSGLGC) that was identical to that reported for *Gloydius blomhoffii* venom [78] (Figure 3). It differed at five residues from the Higuchi *Protobothrops* transcript (Figure 3).

When mass spectrometry was used to analyze crude *Ovophis* venom for the presence of BPPs, the sequence RPPGPPIPP, and derivative forms thereof (PPGPPIPP and GPPIPP) were isolated. This sequence does not occur in our truncated transcript; however, it is nearly identical to a proposed BPP from the N-terminal end of a BPP-CNP transcript from *Gloydius blomhoffii* (RPPGPPIPR) [78,81] and from *Bothrops jararaca* venoms [80] (Figure 3 and Additional file 14: Figure S7).

Potency of bradykinin-potentiating peptides (BPPs) increases ~200-fold if the C-terminal proline residue is doubled [82]. While the C-terminal tripeptide of a BPP from *Gloydius halys* venom was shown to be essential for its activity, removal of the N-terminal pyroglutamate residue made it twice as potent [82]; thus, while the N-terminal pyroglutamate common to BPPs (Additional file 14: Figure S7) may prevent their rapid degradation by prey aminopeptidases, it is actually an impediment to bradykinin potentiation. Interestingly, bradykinin-potentiating activity is not correlated with inhibition of angiotensin-converting enzyme (kininase II) activity [82,83], which is much too slow to be relevant to envenomation.

Various studies have shown that bradykinin potentiation and inhibition of somatic angiotensin-converting enzyme (sACE) by pit viper hypotensive peptides are independent biochemical activities [84-89]. The presence of paired proline residues at the C-terminus and a pyroglutamic acid residue at the N-terminus are not the only requirements for bradykinin-potentiating activity or sACE inhibition. Guerreiro et al. [86] have shown that argininosuccinate synthetase is activated by a BPP from *Bothrops jararaca* venom, indicating that nitric oxide formation represents yet another means by which BPPs promote hypotensive shock to limit prey flight [1].

#### ***Phospholipase B***

**Figure 4:**
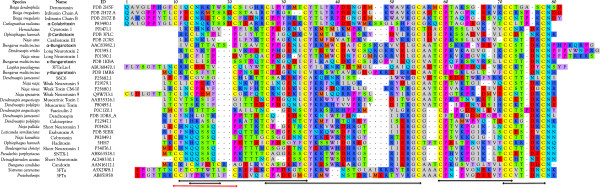
**Alignment of the 5‘ end of the *Protobothrops flavoviridis *phospholipase B (PLB) transcript [AB848155] with the entire *Ovophis okinavensis *PLB transcript [AB848284].** Residues highlighted in orange represent the putative *Crotalus adamanteus* signal peptide sequence [62]. Chymotryptic peptides are shown in purple. Tryptic peptides are in blue. Glu-C peptides are in green. Peptides highlighted in gray were from a venom sample that was undigested. The peptides were naturally occurring, probably as a result of auto-catalysis. Peptide coverage of the *Ovophis* transcript [AB848284] was 50.5%. To the best of our knowledge, these are the first peptidyl data for a snake venom PLB.

Phospholipase B (PLB) activity was first reported in snake venoms by Doery and Pearson [90], who confirmed its presence in the venoms of *Naja naja*, *Pseudechis porphyriacus*, and *Agkistrodon piscivorus*. In 1987, PLB from *Pseudechis colletti* venom was characterized for the first time [91]. No venom PLB sequences were reported until 2011, when transcripts were isolated from venoms of *Drysdalia coronoides*[92] and *Crotalus adamanteus*[62]. While PLB accounted for only 0.06% of all transcripts in those species, it represented 0.14% of *Protobothrops* [AB848155], and 0.15% of *Ovophis* transcripts [AB848284, AB848285] (Additional file 1: Table S1, Additional file 3: Table S2, Additional file 5: Table S3). Peptides covering 26.1% of the *Protobothrops* sequence and 50.5% and 61.6% of the two *Ovophis* sequences, respectively, were isolated by mass spectrometry (Additional file 1: Table S1 and Additional file 3: Table S2; Figure 4). To the best of our knowledge, these are the first protein sequence data for any snake venom PLB.

Feola et al. [93] found that in rabbits, *i.v.* injections of phosphatidylethanolamine (PE) and phosphatidylserine (PS) caused significant hypotension, cardiac arrhythmias, bronchospasm, activation of intravascular coagulation, complement, platelets, and leukocytes with release of histamine, serotonin, and thromboxane at a dose of 0.10 mg/kg and caused cardiac arrest and death at a dose of 0.30 mg/kg. All of these effects are consistent with snake venom envenomation strategies [1]; however, it is not clear whether intact PE and PS are released from cell membranes by pit viper venoms. Kinoshita et al. [94] found that PS and PE were not released from membranes by purified *Protobothrops flavoviridis* phospholipase A_2_; however, one would not really expect this, and venoms contain many other components in addition to phospholipase A_2_. What is more, prey tissue destruction by venom components liberates many endogenous compounds, further complicating the picture. At present, the role of PLB in envenomation remains unclear, beyond its generalized hydrolysis of cell membrane phospholipids.

#### ***Phosphodiesterase***

The *Protobothrops* transcriptome contained four phosphodiesterase (PDE) transcripts, ranging from 0.33-0.56% of all transcripts (Additional file 1: Table S1), which comprised, in aggregate, 0.2% of the transcriptome [AB848150, AB848151, AB848152, AB848153]. Peptides covering 53.4-56.8% of the four PDE sequences were sequenced by MS. PDE was less diversified in *Ovophis* (Additional file 3: Table S2). Two PDE transcripts accounted for a negligible portion of the *Ovophis* transcriptome [AB851989, AB851990]. Sequenced peptides accounted for only 7.8-13.0% of the two PDE sequences.

#### ***Vascular endothelial growth factor-like proteins***

Five VEGF isoforms comprised just over 0.008% of all *Ovophis* transcripts [AB852007, AB852008, AB852009, AB852010, AB848274], while three *Protobothrops* transcripts totaled 0.32% of that transcriptome [AB848141, AB851940, AB851941] (Additional file 1: Table S1, Additional file 2: Table S4, Additional file 3: Table S2, Additional file 4: S5, Additional file 5: Table S3). Fourteen unique peptides were isolated for *Protobothrops* VEGF 1, accounting for 81.1% of its sequence. Fourteen peptides were also sequenced from *Ovophis* VEGF 5, amounting to 60.3% coverage (Additional file 1: Table S1 and Additional file 3: Table S2).

**Figure 5:**
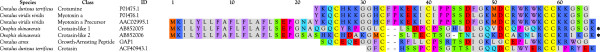
**Alignment of VEGF sequences. Owing to the diversification of this toxin family, classification of venom VEGFs is difficult. ***Ovophis* VEGF 1 [AB852007] possesses a 24-residue insert seen in no other sequence. *Ovophis* VEGF 5 [AB848274] and *Protobothrops* VEGF 1 [AB848141] are homologous to vammin, from the venom of *Vipera ammodytes*. All three of these display short C-terminal extensions of 16-17 residues that bind heparin [102]. Both vammin and VR-1, a VEGF from *Daboia russellii* venom, enhance vascular permeability with great potency. Another subclass of VEGF including *Ovophis* VEGF 3-4 [AB852009, AB852010] and *Protobothrops* VEGF 3 [AB851941] comprise a subclass with no C-terminal extension, or an extremely short extension corresponding to the C-terminus of *Ovophis* VEGF 1-2 [AB852007, AB852008] and *Protobothrops* VEGF 2 [AB851940]. These are significantly shorter than barietin, from the venom of *Bitis arietans*[98], and they do not align well with it or with vammin.

Both venomes contain transcripts for several structural subclasses of VEGFs, although owing to the great diversification of these sequences, classification is difficult. For instance, *Ovophis* VEGF 1 possesses a 24-residue insert seen in no other sequence (Figure 5). *Ovophis* VEGF 1 and 2 and *Protobothrops* VEGF 2 all possess long C-terminal extensions and align well with human VEGF-A_165_ (Figure 5). *Ovophis* VEGF 2 is the most heavily expressed VEGF in that venome, at 0.222% (Additional file 3: Table S2). Human VEGF-A binds to *fms*-like tyrosine kinase-1 (VEGF Receptor-1) (VEGFR-1) and to kinase insert domain-containing receptor (VEGFR-2), but not to VEGFR-3 (fms-like-tyrosine kinase-4) [95-98]. VEGF-A induces vasodilation mediated by nitric oxide [99] and increases vascular permeability 50,000-fold more potently than histamine [100]. In addition, VEGF-A promotes tachycardia, hypotension, and diminished cardiac output when injected *i.v.* in rats [101]. It is likely that *Ovophis* VEGF 1-2 and *Protobothrops* VEGF 2 have similar pharmacology, as these symptoms are consonant with snake envenomation strategies [1].

*Ovophis* VEGF 5 [AB848274] and *Protobothrops* VEGF 1 [AB848141] are homologous to vammin, from the venom of *Vipera ammodytes*. All three of these display short C-terminal extensions of 16-17 residues that bind heparin [102] (Figure 5). Vammin specifically recognizes VEGFR-2 [98]. Both vammin and VR-1, a VEGF from *Daboia russellii* venom, enhance vascular permeability with greater potency than does VEGF-A_165_[98]. Additionally, Yamazaki et al. [103] have shown that a Lys-49 PLA_2_ without catalytic activity further enhances the vascular-permeability promoting capacity of vammin.

*Ovophis* VEGF3-4 and *Protobothrops* VEGF3 comprise a subclass with no C-terminal extension, or an extremely short extension corresponding to the C-terminus of *Ovophis* VEGF 1-2 and *Protobothrops* VEGF2 (Figure 5). These are significantly shorter than barietin from the venom of *Bitis arietans*[98], and they do not align well with it or with vammin (Figure 5).

#### ***5’-Nucleotidase***

Both transcriptomes included a single transcript for 5’-nucleotidase (Additional file 1: Table S1 and Additional file 3: Table S2) [Pf: AB848147; Oo: AB851991]. In both transcriptomes 5’-nucleotidase was a negligible constituent. Mass spectrometry identified 51 venom peptides accounting for 63.3% of the expected sequence of the mature *Protobothrops* protein, while 65 unique peptides were detected in *Ovophis* venom, accounting for 12.9% of the 5’-nucleotidase in that venom.

5’-nucleotidase is ubiquitous in snake venoms [104-107], suggesting a central role in envenomation. This enzyme is known to cleave a wide variety of ribose- and deoxyribose-containing nucleotides [108-110]. It is most active against AMP [109,110] supporting the central role of adenosine in envenomation proposed by Aird [1]. 5’-nucleotidase does not cleave flavin mononucleotide, or cAMP; however, these are hydrolyzed by venom PDE.

#### ***Galactose-binding lectins***

In contrast to C-type lectin-like proteins (CTL), galactose-binding lectins (GBLs) possess intact calcium and galactose-binding loops [72]. GBLs are similar in size to CTL-like proteins and are also dimeric. However, instead of interacting with platelets, GBLs aggregate erythrocytes [111,112]. For this reason, most authors, starting with Gartner et al. [112], have assumed that the presence of GBLs in venom is related to envenomation; however, several lines of evidence raise the possibility of a role unrelated to prey immobilization or digestion [1].

GBLs have been shown to be strongly mitogenic [113-115]. Their mitosis-inducing effects on lymphocytes were found to be comparable to those of concanavalin A [115]. Fry and Wüster [116] noted that GBLs appear to be basal phylogenetically among venomous snakes, whereas CTL-like proteins appear only in the Viperidae. Unlike CTL-like proteins, GBLs display very little sequence variability, suggesting that they are not under selective pressure to diversify, as CTL-like proteins are [117]. Lectins with similar sugar specificity are found in many tissues [118]. In *Protobothrops* and *Ovophis*, GBLs are expressed at very low levels (Additional file 1: Tables S1 and Additional file 3: Table S2) [Pf: AB848130; Oo: AB848277]. Ogilvie et al. [119] likewise found low expression levels for GBLs in *Bothrops atrox* (0.2%) and *Dendroaspis jamesonii* (0.4%) venoms, with a somewhat higher level (0.8%) in *Lachesis muta* venom. Lomonte et al. [120] found that the GBL from *Cerrophidion godmani* venom exhibited edema-forming activity in mice, but concluded that with its low potency and low abundance, it probably plays relatively little role in envenomation. The aforementioned data suggest that GBLs may exist in venom as mitogens to regulate synthetic activity in the glandular epithelium itself. If this view is correct, hemagglutinating and edematogenic activities would be fortuitous, but of secondary importance. Nonetheless, the relative importance of such activities might vary among taxa.

#### ***Aminopeptidases***

Aminopeptidase N plays a significant role in preventing hypertension by degrading Angiotensin III to Angiotensin IV [121]. The role of aminopeptidase A in blood pressure regulation appears to be more complex. APA degrades Angiotensin II to Angiotensin III [122]. When acting at peripheral sites, Angiotensin III is less potent hypertensive than Angiotensin II [122], but in central sites, Angiotensin III raises blood pressure more effectively than Angiotensin II [122,123].

Various lines of evidence suggest a role for APA in promoting hypotension in situations analogous to envenomation. Systemic administration of APA in spontaneously hypertensive rats [124] or hypertensive rats infused with angiotensin II [125] reduced their blood pressure. Administration of amastatin, an APA inhibitor, raised blood pressure in normotensive rats [126].

To date nominal aminopeptidases A and N have been isolated from pit viper venoms, although expression levels appear to be generally low, and many venoms apparently may not contain either. In the present study, *Ovophis* venom contained a complete transcript for Aminopeptidase A [AB848288], while *Protobothrops* venom contained two APA transcripts [AB848148, AB848149]. However, the *Ovophis* Aminopeptidase A transcript comprised only 0.002% of all transcripts, while the two more abundant *Protobothrops* transcripts together comprised 0.073%; hence both are very minor venom constituents. *Ovophis* APA and *Protobothrops* APA 1 were closely related to that reported from *Gloydius brevicaudus* venom [127], differing at only 24 and 22 residues out of 953, respectively.

Tu and Toom [128] found that nearly all venoms hydrolyze L-leucyl-β-naphthylamide, but that there exists great variation in activity levels. Aird [1] suggested that the principal function of leucine aminopeptidase (arylamidase) (LAP) is digestive and that it links the hemorrhagic venom metalloproteases and other venom and endogenous prey peptidases, to L-amino acid oxidase in order to potentiate H2O2 liberation, resulting in hypotension and anticoagulation. It is probable that numerous other amino- and carboxypeptidases in plasma also pass free amino acids to LAO. Clearly the release of Leu from circulating peptides is not solely dependent upon venom LAP. This may partly explain the variation in LAP levels that exists among different venoms [107]. If LAP is abundant in prey tissues, there may not be great selection pressure governing its level of expression in venoms. In the two transcriptomes, LAP was a very minor component [Pf: AB851938; Oo: AB851994] (Additional file 2: Table S4 and Additional file 4: Table S5).

The *Protobothrops* transcriptome possessed two aminopeptidases that show similarity to Aminopeptidase N [AB851954, AB851955] (Additional file 2: Table S4), but some of these did not manifest much similarity to the two *Gloydius brevicaudus* enzymes [127]. They also showed similarity to Aminopeptidase A, so without careful biochemical analyses it is impossible to classify them precisely. Furthermore, it may be that the aminopeptidase nomenclatural system devised for use with human enzymes, may not be applicable to snake venom aminopeptidases.

#### ***Dipeptidyl peptidase IV***

Dipeptidyl Peptidase IV (DPP IV) was first discovered in venoms of various *Micrurus* species by Jorge da Silva and Aird [107]. It was also detected in the venoms of two other elapids, *Bungarus multicinctus*, *Naja naja*, and in that of the Brazilian crotaline, *Bothrops moojeni*. DPP IV titers varied by more than 4x among the different venoms. DPP IV is believed to function in envenomation by blunting a hypertensive response on the part of envenomated prey [1]. Ogawa et al. [129] published the first snake venom DPP IV primary structures, a pair of isomeric sequences derived from cDNA libraries of *Gloydius brevicaudus* venom glands. They determined that the signal peptide was not removed from these sequences. Later Ogawa et al. [130], showed that DPP IV, is actually secreted membrane-bound in exosomes. These micro-vesicles probably account for the “pre-peak” that elutes well ahead of the largest proteins when snake venoms are fractionated using gel filtration chromatography [131,132]. Exosomes were later shown to be present in human saliva as well [133]. DPP IV is nearly ubiquitous among elapid and viperid venoms, but it exhibits great quantitative variability even among full siblings [134].

The *Protobothrops flavoviridis* DPP IV sequence [AB851922] comprises 751 residues, like those from *Gloydius*, while the *Ovophis* sequence has 752 [AB848286]. Nonetheless, the *Protobothrops* and *Ovophis* sequences are more similar to each other than to the *Gloydius* sequences (Additional file 15: Figure S8). The *Protobothrops* sequence is missing one of a pair of asparagine residues present in the other three sequences, but both the *Protobothrops* and *Ovophis* sequences have a leucine residue that is missing in the *Gloydius* sequences (Additional file 15: Figure S8). No DPP IV peptides were discovered with mass spectrometry following enzymatic digestion of *Protobothrops* venom; however, three unique peptides accounting for 4.6% of the *Ovophis* DPP IV sequence were isolated. Venoms were well centrifuged before sample digestion, which probably pelleted the exosomes; thus it is surprising that any *Ovophis* peptides were identified.

#### ***Glutaminyl cyclase***

QC cyclizes, and thereby protects the N-termini of biologically active peptides, such as the BPPs [135], some metalloproteases [136-138], and the B and C chains of the acidic subunit of crotoxin homologs [139,140]. No direct role in envenomation has been suggested for QC to date. However, while cyclization protects these peptides against degradation by prey plasma aminopeptidases, in the case of BPPs, bradykinin-potentiating potency is reduced by half [82].

A total of five snake venom QC cDNAs have been sequenced to date. Two of these belong to colubrids of the Genus *Boiga*[141] and the other three have been sequenced from crotalids on three different continents (*Gloydius blomhoffii*, *Bothrops jararaca*, and *Crotalus adamanteus*).

**Figure 6:**
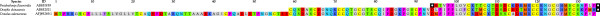
**Alignment of four *Protobothrops *and two *Ovophis *glutaminyl cyclase (QC) sequences with bovine QC and with sequences reported from two colubrid and three additional crotalid venoms.** The two long *Protobothrops* transcripts [AB848133 and AB848134] show near identity with other crotalid sequences, except for an N-terminal 15 residues upstream of the N-terminal methionine. The short *Protobothrops* sequences [AB851933, AB851934] are missing the N-terminal 37 residues of the longer sequences. The next eight residues of the short sequences (QC 3-4) are unique, but thereafter they are identical to the long sequences. *Ovophis* venom also contains two QC [AB852014, AB852015] sequences, but owing to the lack of an N-terminal stop codon, no conclusions can be drawn regarding their length. Positions 18 and 47 differentiate *Boiga* from the crotalids. Positions 27, 294, 298, and 300 are variable across the different taxa.

The present study adds eight additional sequences, of which a couple are distinctly different from those previously published. The *Protobothrops* sample contained four QC transcripts for two pairs of toxins [AB848133, AB848134, AB851933, AB851934]. The two identical long *Protobothrops* transcripts show near identity with other published crotalid sequences (Figure 6). However, as confirmed by the presence of stop codons, two other identical short sequences are missing the N-terminal 37 residues of the longer sequences. The next eight residues of the short sequences are unique, but thereafter they are identical to the long sequences (Figure 6). Pawlak and Kini [141] reported a similar, though less extensive deletion in the *Boiga dendrophila* QC; thus it is clear that this sort of alternate splicing/post-translational modification is characteristic of snake venom QCs. *Ovophis* venom also contains four QC sequences [AB852014, AB852015, AB851985, AB851986], but because all are incomplete, no conclusions can be drawn regarding their length. The most highly expressed of these four represented only 0.008% of all transcripts (Additional file 3: Table S2), consistent with an indirect role in envenomation. Peptides were isolated for all four *Protobothrops* QCs, but only one of the *Ovophis* isoforms.

#### ***Hyaluronidase***

Hyaluronidase is not a major constituent of either venom. A single complete transcript was found in the *Protobothrops* library [AB851937], while two complete *Ovophis* transcripts were sequenced [AB851977, AB851978]. No hyaluronidase transcript was more abundant than the cutoff for contaminants and no peptides were isolated from either venom. Venom hyaluronidase has been deemed a “spreading factor” because its degradation of the extracellular matrix enables other venom constituents, such as metalloproteases and phospholipases, to attack additional tissues [142,143]. As such, hyaluronidase probably serves primarily to digest the prey.

#### ***Three-finger toxins***

**Figure 7:**
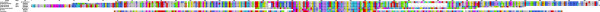
**Alignment of the *Protobothrops flavoviridis *three-finger toxin [AB851958] with selected sequences from colubrid, elapid, and viperid venoms.** Not surprisingly, the *Protobothrops* sequence aligns most closely with that from *Sistrurus*, one of only two other crotalid 3FTx sequences available to date. Both the *Protobothrops* and *Sistrurus* sequences share an N-terminal sequence, EPGYT; however, the first two Cys residues in the *Sistrurus* sequence occur in positions 8 and 11, whereas the *Protobothrops* sequence has paired Cys residues at positions 8 and 9. The *Protobothrops* sequence also has a Pro residue in position 12 which does not occur in the *Sistrurus* mRNA. Based upon the mapping of disulfide bonds in candoxin, the elapid 3FTx most similar to the crotalid toxins [145], it appears that in the *Sistrurus* toxin Cys-11 is bonded to Cys-18, whereas in the *Protobothrops* toxin, Cys-9 is bonded to Cys-18 (red arrow), but these tentative assignments require experimental confirmation. All other disulfide bonds are indicated with black arrows.

*Protobothrops* venom, but apparently not that of *Ovophis*, contains a three-finger toxin (3FTx) [AB851958]. This sequence is most closely related to a transcript reported from *Sistrurus catenatus edwardsi* venom [144] and to candoxin isolated from the venom of an elapid, *Bungarus candidus*[145] (Figure 7). 3FTxs were not detected in an earlier study of *Sistrurus catenatus barbouri* venom [146], and they have not been observed in many other venomics studies of pit vipers [62,147-152]. Other studies have located 3FTxs by transcriptomic means, but not by proteomics approaches [15]. This is not surprising, given their low expression levels in many taxa (0.8% in *Sistrurus catenatus* venom [144]). While 3FTxs are minor components of most pit viper venoms, relatively high expression levels have been reported in some species. In a study of Caribbean pit vipers, using Roche 454 sequencing technology, Durban et al. [32] reported considerable variability (*Crotalus simus*, 12.7%, western *Bothrops asper*, 4.7%; *Bothriechis schlegelii*, 3.6%; eastern *Bothrops asper*, 0.8%; *Cerrophidion godmani*, 0.6%; and *Atropoides picadoi*, 0.4%).

The *Protobothrops* 3FTx differs slightly in its disulfide bond structure from all known 3FTxs (Figure 7). It shares a cysteine residue in position 18 with the 3FTx from *Sistrurus catenatus edwardsi* venom; however, Cys-11, which is linked to Cys-18 in the *Sistrurus* toxin, in the *Deinagkistrodon acutus* short neurotoxin, and in candoxin, occurs at position 9 in the *Protobothrops* toxin (Figure 7).

#### ***Enzymes involved in purine and pyrimidine biosynthesis***

Aird [1] explained the neuromodulatory and hypotensive roles of purine nucleosides in the pharmacology of snake envenomation. A later study quantified purine and pyrimidine nucleosides in a wide variety of elapid, viperid, and crotalid venoms [31]. Possible roles of uridine and cytidine in envenomation are less clear than those of purine nucleosides. Because nucleosides are endogenous regulatory substances in all vertebrates, it is impossible for any prey species to develop resistance to them; thus they represent the perfect predatory biochemical weapon. However, their endogenous nature also means that the enzymes involved in nucleoside biosynthesis would be expected in any venom gland transcriptome, regardless of whether nucleosides are actually secreted into the venom in quantities relevant to envenomation. As a result, no venomics studies to date have specifically looked for the presence of nucleoside biosynthetic enzymes. Instead they have been treated as “housekeeping” genes. In fact, only Rokyta et al. [62] have reported the sequences of adenylosuccinate synthetase, adenylosuccinate lyase, IMP dehydrogenase, GMP synthetase, nucleoside monophosphate kinase, nucleoside diphosphate kinase, or CTP synthetase.

In both transcriptomes, we found transcripts for all four of the enzymes required to synthesize AMP and GMP from IMP [adenylosuccinate synthetase, Pf: AB851944; Oo: AB851992, AB851995; adenylosuccinate lyase, Pf: AB851928; Oo: AB851974; IMP Dehydrogenase, Pf: AB848116; Oo: AB851975, AB851979, AB852003; GMP synthetase, Pf: AB851932, AB851936, AB851946, AB851952; Oo: AB851972, AB851983, AB851999] (Additional file 1: Table S1 and Additional file 2: Table S4). The monophosphates may then be dephosphorylated by a variety of non-specific phosphatases or by venom or endogenous prey 5’-nucleotidase. Regarding pyrimidine biosynthetic enzymes, nucleoside diphosphate kinase [Pf: AB851920, AB851929; Oo: AB851996, AB852013] and CTP synthetase [Pf: AB851957, AB851927, AB851931; Oo: AB851984, AB851993] were found in both transcriptomes, but nucleoside monophosphate kinase was detected only in *Protobothrops* [AB851951](Additional file 2: Table S4). All of these sequences were identical or nearly so to those reported by Rokyta et al. [62,153].

Unfortunately, because both species in the present study are crotalids, the confirmation of nucleoside biosynthetic enzymes in the venome was less interesting than it might have been. The crotalid envenomation strategy involves liberation of endogenous prey purine nucleosides, but the venoms themselves have a minimal nucleoside content [1,31]. In contrast, some viperid venoms and mamba venoms may contain nearly 9% purines by dry weight [31]. Thus in crotalid venomes, nucleoside biosynthetic enzymes probably are largely metabolic in function. It would be interesting to examine the transcript levels of these enzymes in *Bitis* or *Dendroaspis* venoms by comparison. Direct analysis of venom nucleoside levels would be required to determine what level of mRNA expression corresponds to a departure from metabolic function to envenomation.

#### ***Acid Phosphomonoesterase***

Acid PME comprised a negligible percentage of all transcripts in both venoms (Additional file 2: Table S4 and Additional file 4: Table S5) [Pf: AB851930; Oo: AB851982]. The sequences were most closely related to a tissue PME from *Anolis carolinensis*. To the best of our knowledge, these are the first snake acid PME mRNA sequences reported.

#### ***Acetylcholinesterase***

The *Ovophis* transcriptome included five acetylcholinesterase (AChE) transcripts that collectively amounted to less than the contaminant cutoff for venom gland transcripts, so its presence in the transcriptome may be accidental (Additional file 4: Table S5) [AB852000, AB852001, AB852002, AB851981, AB851973]. AChE activity is considered characteristic of most elapid, but not viperid venoms. AChE transcripts have been reported recently in selected colubrid and dipsadid venoms [11,154]. These are the first reported crotalid transcripts.

#### ***Homologs of crotamine, GAP and crotasin***

Crotamine, a highly basic 42-residue myotoxin was first reported 75 years ago [155] in the venom of *Crotalus durissus terrificus*. Homologs were later discovered in various other rattlesnake venoms [156-160]. These proteins display perplexing geographic distributional patterns [161] and individual quantitative variation [131], and they are products of duplicated loci [162]. Their physiological targets have remained controversial [163-169] and new biochemical activities continue to be discovered [170-176]. Myotoxin *a*, a crotamine homolog from the venom of *Crotalus viridis viridis*, was shown to undergo temperature-sensitive conformational transitions owing to cis-trans isomerization of Pro-20 [177,178]. It is unknown whether the isomers bind to different physiological targets.

Marquardt et al. [179] patented a crotamine homolog called GAP (growth arresting peptide) with mitosis-arresting activity. It was isolated from the venom of *Crotalus atrox*, which, to date, has not been reported to contain a small myotoxin. GAP seems to have gone unnoticed by the toxinological community for the past 24 years, but crotasin, a crotamine homolog with some of the structural features of GAP was reported by Radís-Baptista et al. [180].

**Figure 8:**
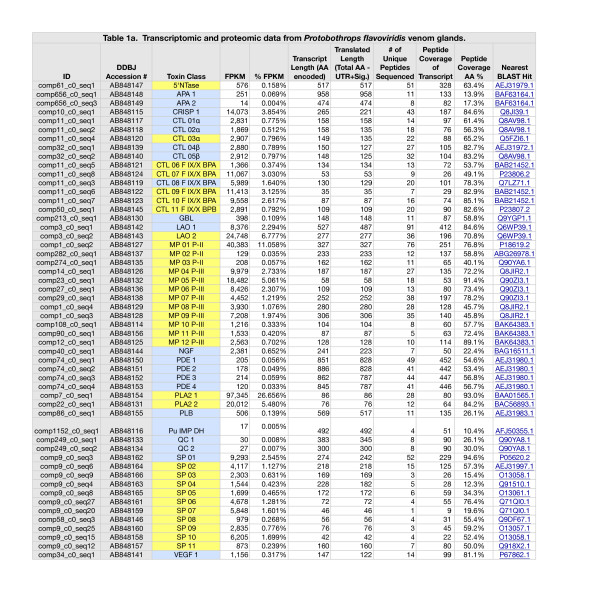
**Alignment of two *Ovophis *transcripts [AB852005, AB852006] with myotoxin a **[156]**and its precursor, crotamine **[155]**, growth-arresting peptide **[179]**, and crotasin **[180]**.** All of these toxins utilize the same scaffold with 35-48 residues and three disulfide bonds. The *Ovophis* transcripts share more features with crotasin and GAP, and they are generally less basic than crotamine and myotoxin a (positions 24, 28, 29, 36, 38, 49, 53, and 55). They lack Met-50 and Trp-54 of the small myotoxins and Trp-56 of the myotoxins and GAP. Both *Ovophis* transcripts bore the sequence RVHGRTSFS upstream from the putative signal peptide (not shown).

The present study isolated two GAP/crotasin-like transcripts from the *Ovophis* transcriptome (Figure 8) [AB852005, AB852006], but no crotamine- or crotasin-like sequence was found in the *Protobothrops* transcriptome. Crotasin/GAP-like proteins are significantly less basic than the crotamine-like proteins, and they lack a Phe-Pro dipeptide (crotamine residues 12-13), as well as the N-terminal Tyr of the latter. The two *Ovophis* transcripts differ very significantly from each other and from both GAP and crotasin (Figure 8). Though the exact location of the N-terminal residue cannot be determined with certainty, they both apparently possess the N-terminal disulfide bond present in crotamine and GAP, but absent in crotasin, and they are comparable in length to crotamine and GAP. Crotasin lacks the N-terminal eight residues of crotamine homologs. However, the signal peptide sequence for various crotamine isomers [181] exactly matches the signal peptide sequences of our *Ovophis* crotasin/GAP homologs. Both *Ovophis* transcripts manifested near-zero transcription levels, so it seems unlikely that these are functional venom components, but it is clear that the sequence diversification that Oguiura et al. [182] reported, applies to these transcripts as well.

#### ***Waprins***

Waprins belong to a family of proteins with diverse activities that are structurally related to whey acidic protein [183]. Other members of the family have anti-bacterial activity and protease inhibitory activity [184,185]. Waprins discovered to date are small proteins of about 50 amino acids, containing four disulfide bonds [186]. Clauss et al. [187] identified a segment of human chromosome 20, displaying 14 genes for proteins related to whey acidic protein. They postulated that the resulting gene products could potentially serve an anti-microbial function against pathogenic bacteria, or that they might participate in the regulation of endogenous proteases. They also opined that kallikrein-like proteases are of particular interest.

The protease inhibitory capacity of members of this family suggests possible roles in envenomation, though to date, no evidence has been presented for any of these functions. Snake venom proteins belonging to the Kunitz/BPTI family have been modified to serve as ion channel inhibitors [188] and to chaperone neurotoxic PLA_2_s [189]. BPPs inhibit angiotensin I-converting enzyme to promote hypotension [83,190], but also may act directly upon other physiological targets to induce hypotension [191,192]. Some of the bradykinin-potentiating peptides serve an interesting dual role by inhibiting hemorrhagic metalloproteases in the venom gland [193].

Pahari et al. [144] reported the first viperid waprin-like protein in the venom glands of *Sistrurus catenatus edwardsi*. However, the putative *Sistrurus* toxin comprised a waprin domain fused to a Kunitz/BPTI domain. The function of the encoded protein is unknown. It was represented by only a single transcript, so it is difficult to say whether this toxin is biologically significant. This non-enzymatic toxin was expressed at near-zero levels.

**Figure 9:**
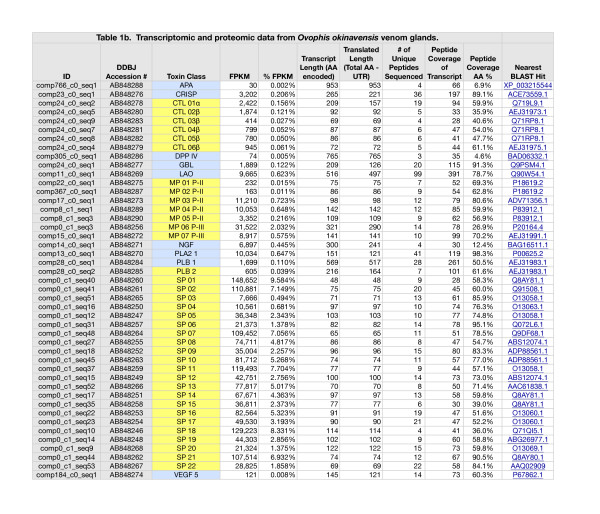
**Alignment of *Protobothrops *[AB851939] and *Ovophis *transcripts [AB852011] with crotalid waprins.** Both transcriptomes contained partial transcripts that were strongly homologous to the *Crotalus adamanteus* waprin.

Rokyta et al. [62] reported a full-length waprin transcript in the venom of *Crotalus adamanteus.* Both the *Protobothrops* and *Ovophis* transcriptomes contained transcripts that were strongly homologous to the *Crotalus* waprin [Pf: AB851939; Oo: AB852011] (Figure 9). Interestingly, the *Ovophis* waprin has a C-terminal Pro-Met, instead of the usual Pro-Leu/Val-Pro. One peptide representing 28% of the transcript sequence was isolated (Additional file 3: Table S2).

Both venoms also contained sequences that are related to the Kunitz serine protease inhibitor domain of the novel ku-wap hybrid toxin from *Sistrurus catenatus edwardsi* venom [62] (Figure 9). All of these transcripts are incomplete and the three N-terminal transcripts show relatively little overlap with the region of fusion in the *Sistrurus* ku-wap toxin; however, all three of the putative ku-wap homologs show the acidic and basic residues (positions 83-84) and other features of the Kunitz domain of the *Sistrurus* toxin (Figure 9). They do not show strong homology to either α-dendrotoxin [194] or to bovine pancreatic trypsin inhibitor (Figure 9). They may be additional examples of the ku-wap family; however, they appear to be most closely related to vertebrate inhibitors of the tissue factor pathway.

#### ***Putative inhibitors of tissue factor pathway***

In vertebrates, blood coagulation is initiated by the tissue factor (TF) pathway. This pathway is regulated primarily by tissue factor pathway inhibitor (TFPI), a Kunitz serine protease inhibitor that inhibits Factor Xa and thrombin at concentrations as low as 2.5 nM, thus controlling the generation of thrombin and ultimately, of fibrin [195,196]. Platelet TFPI is believed to modulate intravascular coagulation [197].

**Figure 10:**
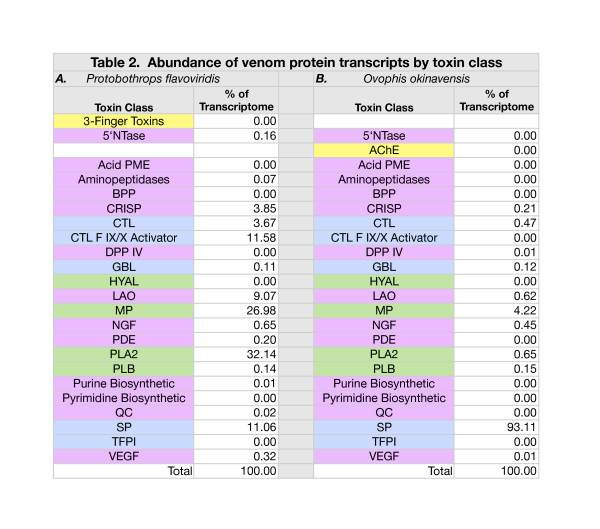
**Alignment of TFPI-like sequences.** The putative *Protobothrops* TFPI transcript [AB851921] is most similar to a DNA sequence from *Anolis carolinensis*. It aligns best at the C-terminus and in the middle, except for a 27-residue deletion in the *Protobothrops* sequence, which separates these two regions. Two partial transcripts from *Ovophis* venom glands [AB851997, AB851998] are identical to that from *Protobothrops* in the middle section. Affinities of these toxins to bovine pancreatic trypsin inhibitor and to the Ku-Wap fusion toxin from *Sistrurus catenatus edwardsi* venom are weak.

The *Protobothrops* transcriptome contained a single, partial transcript [AB851921] and the *Ovophis* transcriptome contained two, very short, identical transcripts [AB851997, AB851998] that align well with a predicted *Anolis* TFPI, and less well with the Ku-Wap fusion toxin from *Sistrurus catenatus edwardsi* venom glands and with bovine pancreatic trypsin inhibitor (Figure 10; Additional file 2: Table S4 and Additional file 4: Table S5). The *Protobothrops* TFPI transcript aligns well with both the acidic N-terminus and the highly basic C-terminus of human TFPIα [198] (Figure 10).

All three transcripts are expressed at vanishingly low levels (~0.001% of all transcripts) and it seems extremely unlikely that they function in envenomation; however, peptides ranging from 6.3% to 11.9% of the *Protobothrops* and *Ovophis* sequences were isolated. Most likely, these are tissue transcripts related to snake vascular homeostasis. If they serve any additional roles, they might inhibit venom SPs in the gland, or they might inhibit prey thrombin, allowing venom SPs to clot fibrinogen improperly, resulting in its rapid clearance by the prey's anti-clotting cascade.

#### ***Paraoxonase***

Paraoxon hydrolytic activity has been reported only in the venom of *Daboia russellii* to date [199]. Venoms of *Naja naja, Crotalus adamanteus,* and *Agkistrodon contortrix contortrix* showed only trace level activity by comparison. Three genes comprise the paraoxonase gene family in humans. PON1 is largely associated with high-density lipoprotein, but has organophosphatase, arylesterase, or lactonase activities, and it hydrolyzes a wide array of substrates [200]. PON2 and PON3 are not well studied, but PON2 is known to be a widely-distributed cellular enzyme. Two transcripts were found in the *Protobothrops* transcriptome, but none in *Ovophis*. Both *Protobothrops* transcripts were expressed at near-zero levels, suggesting that paraoxonase is not a venom component in either of these species. The *Protobothrops* paraoxonase isozymes share diagnostic residues with all three human isozymes and are not clearly related to any one of them [AB851924, AB851925].

#### ***Vespryns***

Pung et al. [201] isolated a novel 12 kDa toxin from the venom of the king cobra that acts centrally to induce hypolocomotion and pain in mammalian prey. A toxin from *Lachesis muta* venom [202] was the first crotalid vespryn and a second was sequenced from *Crotalus adamanteus* venom [62]. The *Protobothrops* transcriptome contained a partial, 70-residue vespryn transcript [AB851949], but the *Ovophis* transcriptome had none (Additional file 16: Figure S9). No vespryn peptides were sequenced. The *Protobothrops* vespryn is most closely related to that from *Lachesis*, which also displays a four-residue gap from positions 25-28. Only three of the first 70 residues differ between these two toxins. The three crotalid vespryns are all 28-32 residues longer at the N-terminus than the two corresponding toxins from *Ophiophagus hannah* and *Pseudechis australis* venoms [203].

## Conclusions

Using two distantly related pit viper species with different venom compositions, our study illustrates the power of using next generation sequencing in combination with LC/MS profiling for the study of venom chemistry. We were able to detect a wide variety of venom components in both cDNA and in the venom itself. Except for the annotation of protein function, the analytic pipeline was entirely self-contained and did not rely on publicly available reference databases. Given the decreasing costs of sequencing, and the increasing power of mass spectrometry, this approach will be increasingly useful for poorly studied species that have no previously published reference data, and also for detecting fundamentally new venom components that might have been missed by earlier investigations.

We show, for the first time, that the composition of venom gland mRNA is linearly correlated with protein composition of the venom. Although this finding is fairly trivial by itself, especially given the amount of unexplained variance observed in our correlation, it has several interesting methodological implications. It appears that peptide detection with LC/MS can potentially be used to quantify individual proteins in venoms. This can allow high-throughput screening of numerous venom samples providing comparative data on the abundance of various components. Although probably not as sensitive or quantitative as cDNA sequencing, at least without further refinement, this approach permits non-invasive sampling, which will be important for rare or endangered species. Crude venom is also easier to collect and store than RNA, making it possible to collect numerous samples in the field, or to use archived venom samples. We are currently conducting studies focused on improving the accuracy of LC/MS-based venom peptide sampling and quantification, and on developing better metrics.

We obtained similarly quantitative results using *de novo* assembled transcriptomes and publicly available data from NCBI for protein identification (Additional file 8: Figure S1). This finding makes mass spectrometry useful even for species without custom-made species-specific reference transcriptomes. Although using publicly available data prevents the discovery of novel proteins, public data should be particularly useful for comparative studies, and for investigation of snakes for which transcriptomes cannot be obtained for whatever reason.

With regard to the utility of using mass spectrometry for non-invasive, quantitative sampling, another pair of studies report the isolation of intact mRNA directly from venoms [204,205]. It remains to be seen how quantitative this technique will prove to be and how useful it will be for archival samples, especially those that have been repeatedly frozen and thawed, but certainly it offers exciting possibilities, especially in combination with mass spectrometry.

The present study reports 103 venom or venom-related cDNA sequences from the venom glands of *Protobothrops flavoviridis*. Of these, 40 were previously known from the literature, although this figure includes isomeric forms not previously reported. Fifty-one sequences were similar to those reported from other venomous snake taxa, but were new for *Protobothrops*. An additional 12 have not previously been reported for any snake. In regard to *Ovophis okinavensis*, 94 of the 95 cDNA sequences reported herein are new for this species, and 13 have not been reported previously for any snake. Peptides were sequenced from 100% of transcripts that were more abundant than contaminants such as human keratin (Additional file 1: Table S1 and Additional file 3: Table S2). Peptides were also sequenced from at least 18 additional transcripts that occurred below the contaminant level.

To the best of our knowledge this study also furnishes the first peptidyl sequence data (peptides sequenced by mass spectrometry) for venom phospholipase B and 5’-nucleotidase, and the first mRNA sequence data for a snake acid PME, adenylosuccinate synthase, paraoxonase, and a putative tissue factor pathway inhibitor. Novel crotasin/growth-arresting peptide/crotamine-like sequences are reported from the *Ovophis* transcriptome. The *Protobothrops* 3FTx sequence is only the third such sequence reported from a crotalid, but it differs in significant ways from the other two sequences.

Dominated by PLA_2_, MPs, and LAO, adult *Protobothrops* venom strategically promotes hemorrhage, hypotension, incoagulable blood, and prey digestion, consistent with mammalian predation. *Ovophis* venom, by contrast, is composed principally of SPs and MPs (93.1% of all transcripts) (Figure 1; Additional file 1: Tables S1, Additional file 3: Table S2, Additional file 5: Table S3). Its composition is less readily interpreted, owing to inadequate pharmacological data for venom proteases. This venom apparently represents a hybrid strategy optimized for frogs and small mammals, but the contributions of most components cannot be unambiguously assessed at present.

## Methods

### Venom and reagents

Venom was extracted from one *Protobothrops flavoviridis* and one *Ovophis okinavensis* at the Okinawa Institute of Health and Environment. Four days later, venom glands were excised from each specimen. Prior to gland removal, the two snakes were anesthetized with chloroform until they showed no righting reflex or tail-retraction reflex. In pit vipers, the tail is always the last part of the body to become anesthetized and the first to recover from anesthesia. This euthanasia protocol complies with the “Guidelines for Proper Conduct of Animal Experiments” (Science Council of Japan, June 1, 2006).

Once the snakes were completely anesthetized, glands and underlying skeletal muscle were quickly excised after dissecting back the overlying skin. Each gland was immediately placed into a pre-labelled 1.5 mL microcentrifuge tube having a screw cap and an O-ring, and dropped into liquid nitrogen. Samples were then stored at -80°C until the following week.

### Isolation of total mRNA from venom glands

Total mRNA isolation employed a Qiagen RNeasy Plus Mini Kit and utilized the following procedure. Glands were removed from storage and their masses were determined without allowing them to thaw. Glands were immediately dropped into a 50 mL Falcon tube containing 3 mL of Buffer RLT Plus containing 1% β-mercaptoethanol. Additional buffer was added after homogenization was begun. Ideally 600 μL of buffer should be used for every 30 mg of tissue. Accordingly, the 350 mg *Protobothrops* gland was homogenized in 6.5 mL RLT buffer, but because the *Ovophis* gland weighed just 100 mg, only 2 mL were needed, but in the interest of prompt homogenization, 3 mL were used anyway.

Lysates were centrifuged 3 min at maximum speed and 600 μL were transferred to each of five gDNA Eliminator spin columns. All ten samples were then processed according to Qiagen’s instructions. Eluents from the five tubes were pooled for each of the samples.

Next the Ambion LiCl RNA precipitation technique was employed, after reserving 50 μL of each pool for analysis on the Nanodrop ND-1000. Pellets were resuspended 10 mM Tris, 1 mM EDTA.

All four samples were diluted to bring them into the 25-500 ng/μL range for analysis on an Agilent Bioanalyzer 2100 using an RNA Nano 6000 chip. The pre-LiCl *Protobothrops* sample had an RNA Integrity Number (RIN) of 9.5, while the other three samples were all 10.0.

### cDNA synthesis and preparation of Illumina RNA-Seq libraries with barcodes

Post-LiCl samples were used for first strand cDNA synthesis. In 200 μL PCR tubes, 1 μL of each total RNA sample was combined with 3 μL water and 1 μL of 10 μM Cap-TRSA-CV primer (AAGCAGTGGTATCAACGCAGAGTCGCAGTCGGTACTTTTTTCTTTTTTV). Samples were incubated 3 min at 65°C, and then chilled on ice. Total RNA concentrations for the *Protobothrops* and *Ovophis* samples were 1,282 and 930 ng/μL, respectively.

Next the following were added to each tube: 2.0 μL 5x first-strand synthesis buffer (Clontech ST0079), 0.5 μL 10 mM dNTP (Clontech ST0073), 1.0 μL 0.1 M DTT (Invitrogen Y00147), 1.0 μL 10 μM template-switch primer (RNA oligo Smarter IIA, Clontech ST0069), and 1 μL Superscript II reverse transcriptase (Invitrogen). Tubes were incubated 1 hr at 42°C. Reactions were terminated by heating at 65°C for 15 min. Tubes were then placed on ice and samples were diluted with 40 μL water prior to cDNA amplification.

Eight tubes of each first-strand cDNA were prepared for second-strand synthesis and amplification using an 8.5x master mix containing: 25.5 μL first-strand cDNA, 178.5 μL water, 25.5 μL 10x PCR buffer (10x Advantage 2 SA PCR Buffer (S3245), 6.375 μL 10 mM dNTP, 11.9 μL cDNA Amplification primers (Clontech Nested Universal primer A, ST0102), and 5.1 μL Advantage 2 polymerase (Clontech).

Using a thermocycler, samples were heated to 95°C for 1 min. This was followed by 11 cycles of (95°C for 10 sec/68°C for 6 min). Then the temperature was reduced to 72°C for 10 min, before cooling to 4°C. PCR products were purified with a QIAquick PCR purification kit (Qiagen). Products were analyzed on a Nanodrop ND-1000 to determine double-stranded cDNA concentrations.

Eight μL of each purified sample were loaded into a 1% agarose gel and electrophoresis was performed in 1x sodium borate buffer (56.6 mM Boric acid, pH 7.5 adjusted with NaOH) at 100 V for 30 min. New England Biolabs 2-log DNA Ladder (0.25 μL) was used to estimate DNA size.

Tagmentation followed the Epicentre Nextera DNA Sample Prep Kit (Illumina-compatible) protocol in a one-third size (6.7 μL) reaction volume. The following components were assembled on ice: 4.2 μL and 4.65 μL nuclease-free water (for the *Protobothrops* and *Ovophis* samples, respectively), 16.7 ng target DNA in 10 mM Tris-HCl (pH 7.5) with 1 mM EDTA, 1.35 μL 5X Nextera reaction buffer HMW, 0.35 μL Nextera enzyme mix (Illumina-compatible). The above reaction mixture was briefly vortexed, and incubated at 55°C for 5 min in an MJ Research PTC-200 peltier thermocycler with a heated lid. Tagmented DNA was purified using the Qiagen Min Elute protocol. We used Buffer ERC in the MinElute Reaction Cleanup Kit because it efficiently binds double-stranded DNA ≥ 70 bp and removes enzymes, salts, and oligomers. The final step was to add DNA barcodes and to enrich the library following the Epicentre Nextera DNA Sample Prep Kit (Illumina-compatible) protocol.

### Sequencing and bioinformatics

Both libraries were sequenced in a single Illumina GAII lane using 75-bp paired-end reads at OIST’s sequencing center, according to the manufacturer’s specifications. After quality filtering with Condetri (v. 2.0) [206] using the default setting, the reads were assembled using the Trinity RNA-seq suite [207,208]. FPKM values for the isoforms were computed using the RSEM package [209] included with Trinity. Using a threshold recommended by Mortazavi et al. [210], we filtered low abundance transcripts with FPKM less than 1, and used these as reference sequences for the proteomic pipeline.

### Reduction, alkylation, and digestion of venoms with trypsin and chymotrypsin

Crude venom was centrifuged 10 min at maximum speed (20,238 rcf). Reactions were performed in 200 μL PCR tubes. Reduction was accomplished using a reaction mixture that contained 37 μL ultrapure water, 1 μL venom (200-300 μg protein), 2 μL 500 mM DTT in ultrapure water, and 10 μL 500 mM Tris-HCl (pH 8.0). Tubes were incubated 45 min at 60°C in the dark in a thermocycler. Following venom protein reduction, 10 μL (0.38 mg) of iodoacetic acid Na^+^ salt (Nacalai 19305-24) in ultrapure water were added to each tube and mixed with pipetting and gentle vortexing. Tubes were incubated 30 min at 37°C in the dark. Then 1 μL of 500 mM DTT was added to quench the alkylation reaction. Next 4.5 μL of 200 mM CaCl_2_ (Nacalai 06731-05) were added to each tube. An additional 5 μL of 500 mM Tris-HCl (pH 8.0) were added to maintain the pH and ionic strength. Finally, 10 μg of trypsin (Pierce 90055) or chymotrypsin (Pierce 90056) dissolved in 1 mM HCl were added to each tube. Tubes were incubated 24 h at 37°C and then frozen at -30°C until preparation for mass spectrometry.

### Digestion of venoms with Glu-C

Reduction and alkylation of venoms were performed as described above, except that instead of 500 mM Tris-HCl, 167 mM phosphoric acid/NaOH (pH 8.0) was used. Furthermore, the enzyme was dissolved in ultrapure water, rather than in 1 mM HCl. This enabled the enzyme to cleave proteins adjacent to aspartic acid residues, as well as glutamate residues. When the enzyme was dissolved in 1 mM HCl, it cleaved next to glutamate residues only, despite the use of phosphate buffers for hydrolysis.

Unlike trypsin and chymotrypsin, Glu-C (Pierce 90054) was inhibited by iodoacetate. It was necessary to desalt the reaction mixture before enzymatic digestion. Desalting was accomplished using Zeba Spin Desalting Columns (0.5 mL, Pierce 89882). Because naturally-occurring small peptides in venoms, such as bradykinin-potentiating peptides are removed by these spin columns, samples of crude venoms were also prepared for direct analysis by mass spectrometry, after removal of large proteins.

### NanoLC-mass spectrometric analysis

A Thermo Scientific LTQ Orbitrap hybrid mass spectrometer was used for MS data collection. The mass spectrometer was equipped with an HPLC (Paradigm MS4, Michrom Bioresources, Inc.), an autosampler (HTC PAL, CTC Analytics) and a nanoelectrospray ion source. Each venom digest was desalted using a ZipTip C_18_/P10 (Millipore) prior to the NanoLC-MS run. Clean sample was separated on a capillary reverse phase column (50 × 0.15 mm, 3 μm, MS C_18_, Grace Vydac). A one-hour gradient (10% B to 30% B in 60 min, where solvent A is 2% acetonitrile and 0.1% formic acid, and solvent B is 98% acetonitrile and 0.1% formic acid, flow rate 2.0 μL/min) was used for the peptide separation. The temperature of the heated capillary was 200°C, and 1.70 kV spray voltage was applied to all samples. The mass spectrometer’s settings were, full MS scan range 350 to 1500 m/z, with mass resolution of 60,000 at 400 m/z, 50 μs scan time with accumulation of three microscans. The three most intense ions from this full MS scan were fragmented in data-dependent manner with CID, using an exclusion list of 500 ions during 30 seconds. Triplicate NanoLC-MS analyses were run for every venom digest sample.

### Protein Identification

Analysis of mass spectrometric data was performed using three different search engines: Mascot (version 2.4), Proteome Discoverer (version 1.2) and PEAKS (version 4.2 SP 1). Fragmentation spectra were filtered using Proteome Discoverer, allowing only double to quadruply charged ions, and removing the precursor ion within a window of 1 Da. Processed spectra were searched using Sequest and Mascot. Two missed cleavages were allowed, and precursor and fragment mass tolerance were set to 20 ppm and 0.8 Da, respectively. Carboxyamidomethylation of cysteine was set as a fixed modification, while methionine oxidation and asparagine and glutamine deamidation were set as variable modifications. Enzymes used for sequencing (trypsin, R and K; chymotrypsin, F, L,W, and Y; Glu-C, D and E) were specified in each case. For naturally occurring peptides (undigested venom samples), no enzyme was specified in the search. A constructed database, using the six possible frames for each detected transcript, with the common Repository of Adventitious Proteins – cRAP (http://www.thegpm.org/crap/) was used for both search algorithms (*Protobothrops* plus cRAP = 20,945 entries; *Ovophis* plus cRAP = 15,264 entries). Protein and peptide identifications from Mascot and Sequest results were combined, setting the false discovery rate to 1%.

Spectra not identified were submitted for de-novo sequencing using PEAKS. Search parameters were the same as defined for Mascot and Sequest, except for specifying the mass spectrometer as an FT-trap, and allowing ≤3 modifications per peptide. Results were filtered to allow only sequences with rank equal to zero and a PEAKS score higher than 20. These sequences were BLASTed against our constructed databases, and filtered, allowing only matches with an E score <0.05.

Combined results of all three search engines were used to report protein and peptide identifications. The same search (using Mascot and Sequest only) was performed using the NCBI database, subset for snake taxonomy (txid8570; 40,887 sequences).

### RNA-seq and proteomic comparisons

Because longer transcripts produce more fragments, RNA-seq data are typically analyzed using metrics which standardize the number of reads mapped to a particular exon by the total number of mapped reads and the size of the exon [210]. We attempted an analogous measure of protein abundance based on peptides, to prevent longer proteins from appearing more abundant than they are. Unlike mRNA reads, each of which competes for a position in the flow cell, with adequate chromatographic separation, peptides are detected sequentially during their elution from the liquid chromatograph, and should be detected independently of one another. Under this assumption, we did not standardize by the total number of detected fragments. For each protein identified, we counted the total number of peptide fragments. Then we divided this number by the length of the protein to standardize for size, producing a measure of peptides per unit length of protein, which could then be correlated with the FPKM metric, computed as described above. The count of each peptide mapping to different proteins was divided by the number of matches, to account for mapping uncertainty.

To evaluate the robustness of our analysis relative to the reference protein data set chosen, a separate analysis was conducted using snake venom proteins from the publicly available NCBI database, for protein identification. This analysis was conducted as described above, except that that PEAKS identification was omitted in the interest of time. We used reciprocal best BLAST as the criterion for establishing homology between NCBI data and the *de novo* sequenced transcriptomes. This was a conservative choice, since many isoforms or closely related genes could generally have just one NCBI best hit.

The cRAP protein database, which lists common contaminants, was used to determine abundance thresholds for including predicted proteins. To determine this cutoff, we bootstrapped the 99.9% confidence intervals around the abundance scores for human contaminant proteins, which were most likely introduced during sample preparation, and which should be present at much lower concentrations than target proteins. Proteins below this threshold were filtered from the analysis.

## Competing interests

The authors declare that they have no competing interests.

## Authors’ contributions

This project was conceived and planned by SDA and ASM. All authors participated in data collection. KT obtained, maintained and furnished the snakes. SDA and YW created the cDNA library. MCR performed pilot mass spectrometric data analyses, in addition to processing all of the mass spectrometry samples. AVB designed and revised the mass spectrometric techniques, wrote scripts to extract and process data, and summarized peptidyl data for subsequent comparisons. ASM processed transcriptomic and proteomic data, devised measures of peptide abundance, and performed statistical analyses. SDA reviewed the toxinological literature and analyzed transcriptomic and proteomic data in relation to venom chemistry. SDA and ASM wrote the manuscript. All authors read and approved the final manuscript.

## Supplementary Material

Additional file 1: Table S1 Abundance of individual toxin transcripts in the *Protobothrops flavoviridis* transcriptome, as RNA Fragments/Kilobase of Transcript Sequence/Million Base Pairs Sequenced (FPKM), arranged by toxin class. Transcripts that were less abundant than contaminant levels (e.g. human keratin) were not included in this table, even in cases in which peptides corresponding to those transcripts were isolated. Transcripts in blue are complete while those in yellow are incomplete. All significant venom constituents were identified by mass spectrometry. The number of amino acid residues and the percent coverage of the predicted protein by sequenced peptides are also shown. The expected protein length was determined from the transcript length minus untranslated regions and the putative signal peptide, if any. Click here for file

Additional file 2: Table S4 *Protobothrops flavoviridis* transcripts that had negligible FPKMs. Incomplete transcripts are highlighted in yellow; complete transcripts are shown in blue. Peptide coverage data are presented for those transcripts with sequenced peptides. There is a high degree of certainty associated with all sequences except those highlighted in gray, although they may also be valid. Click here for file

Additional file 3: Table S2 Abundance of individual toxin transcripts in the *Ovophis okinavensis* transcriptome, as RNA Fragments/Kilobase of Transcript Sequence/Million Base Pairs Sequenced (FPKM), arranged by toxin class. Transcripts that were less abundant than contaminant levels (e.g. human keratin) were not included in this table, even in cases in which peptides corresponding to those transcripts were isolated. Transcripts in blue are complete while those in yellow are incomplete. All significant venom constituents were identified by mass spectrometry. The number of amino acid residues and the percent coverage of the predicted protein by sequenced peptides are also shown. The expected protein length was determined from the transcript length minus untranslated regions and the putative signal peptide, if any. *In the *Ovophis* transcriptome, an incomplete transcript for bradykinin-potentiating peptides and C-type natriuretic peptides was isolated. A single peptide was sequenced by mass spectrometry, but based upon a BLAST search, it originated in the missing portion of our transcript; hence coverage is given as 0%. Click here for file

Additional file 4: Table S5 *Ovophis okinavensis* transcripts that had negligible FPKMs. Incomplete transcripts are highlighted in yellow; complete transcripts are shown in blue. Peptide coverage data are presented for those transcripts with sequenced peptides. There is a high degree of certainty associated with all sequences except those highlighted in gray, although they may also be valid. ***One peptide (RPPGPPIPP) and two derivatives of the *Ovophis* BPP sequence were sequenced by mass spectrometry. This sequence does not occur in our truncated transcript, so no percent coverage is given; however, it is nearly identical to a proposed BPP from the N-terminal end of a BPP-CNP transcript from *Gloydius blomhoffii* (RPPGPPIPR) [78,81] and from *Bothrops jararaca* venoms [80]. Click here for file

Additional file 5: Table S3 Abundance of venom protein transcripts by toxin class in *Protobothrops flavoviridis* and *Ovophis okinavensis* venoms. The different envenomation strategies employed by these two pit vipers differ significantly, as illustrated especially by expression levels of MPs, PLA_2_s, SPs, and CRISPS. Color fills indicate primary functions of toxin classes; however, many venom components have direct or indirect secondary and even tertiary functions. Yellow: Neurotoxic; Pink: Hypotensive; Blue: Anticoagulant; Green: Digestive. Some toxin functions are strategically counterintuitive. For example, thrombin-like SPs are directly pro-coagulant, but by activating the prey fibrinolytic system, their ultimate effect is anticoagulant. Click here for file

Additional file 6: Table S6 Peptide coverage of non-venom-related transcripts from the cDNA libraries of *Protobothrops flavoviridis* venom glands. “Adjusted Counts” were used to discount peptides that matched multiple proteins, so as to avoid spuriously high values. Adjusted counts were used to create Figure 2 and Additional file 8: Figure S1. Click here for file

Additional file 7: Table S7 Peptide coverage of non-venom-related transcripts from the cDNA libraries of *Ovophis okinavensis* venom glands. “Adjusted Counts” were used to discount peptides that matched multiple proteins, so as to avoid spuriously high values. Adjusted counts were used to create Figure 2 and Additional file 8: Figure S1. Click here for file

Additional file 8: Figure S1 Correlation between abundances of proteins predicted using NCBI data (black) and *de novo* assembled reference sequence (grey). Homologies between the two protein sets were determined using reciprocal best BLAST, so many of the proteins detected in the *de novo* transcriptome were omitted in the comparison, because they did either did not have homology to known snake proteins, or this relationship could not be determined with certainty, *e.g.*, in the case of multiple isoforms or closely related genes. Nonetheless, the correlation coefficients were close between the two data sets, suggesting that the measure of protein abundance was robust to the choice of protein reference data set (*Protobothrops*: NCBI r = 0.52, p = 0.014, Trinity r = 0.64, p = 2.2e^-16^; *Ovophis*: NCBI r = 0.64, p = 1.2e^-4^, Trinity r = 0.68, p = 6.3e^-10^). Note that the correlation coefficients differ slightly with Figure 2, since the analysis presented in Additional file 8: Figure S1 did not involve assignment of unmapped proteins by PEAKS. Click here for file

Additional file 9: Figure S2 Alignment of metalloproteases from the *Protobothrops flavoviridis* transcriptome. These sequences assort into two distinct groups, upper and lower. Members of the lower group display significant similarities and align well. Members of the upper group, for the most part, align poorly with one another, and essentially not at all with the lower group. Both groups contain both P-II and P-III MPs. Given the size of many MPs, some of these partial sequences probably represent non-overlapping segments, despite attempts by the software to align them. Click here for file

Additional file 10: Figure S3 Alignment of metalloproteases from the *Ovophis okinavensis* transcriptome, showing the sequences of five P-II and three P-III MPs. Click here for file

Additional file 11: Figure S4 Alignment of 18 serine protease sequences from the *Protobothrops flavoviridis* transcriptome. SP12 appears to be an inactive plasminogen activator transcript, while SP11 is probably a truncated member of the same subclass. Click here for file

Additional file 12: Figure S5 Alignment of 26 *Ovophis okinavensis* serine protease sequences. It is impossible to infer biological activities from these transcripts; however, the *Ovophis* transcripts seem to fall into three or four structural subclasses or groupings. SP15 and related sequences with clusters of three acidic residues (positions 121-123) and three aromatic residues (position 132-134) appear most similar to thrombin-like enzymes. SP05 and 06 all display a high percentage of aliphatic and aromatic residues (positions 116-140), but their biological activity is not known. SP08 is apparently a thrombin-like enzyme. SP09 is most similar, based on this fragment, to an SP from *Protobothrops jerdonii* venom that has lost two of the three catalytic residues of active SPs. Click here for file

Additional file 13: Figure S6 Alignment of all CTL transcripts from both venoms with sequences of convulxin (*Crotalus durissus terrificus*) and flavocetin (*Protobothrops flavoviridis*). *Protobothrops* venom contained various Factor IX/X binding proteins that were absent in *Ovophis* venom. Click here for file

Additional file 14: Figure S7 Alignment of known bradykinin-potentiating peptides from various viperid venoms showing the great sequence variability in this toxin class [78-83,191,211-222]. Click here for file

Additional file 15: Figure S8 Alignment of *Protobothrops flavoviridis* [AB851922] and *Ovophis okinavensis* [AB848286] dipeptidyl peptidase IV sequences with two isomers from *Gloydius brevicaudus* venom. The former sequences each possess a leucine residue in position 268 that is missing in the *Gloydius* sequences. They also have Gly-80 where *Gloydius* has Glu, Ile-85/Val, Asn-113/Ser, Thr-170/Ala, Ser-215/Arg, Ala-395/Ser, Arg-502/Ser, Gly-632/Asp, and Glu-680/Lys. The *Protobothrops* sequence lacks asparagine-133, which is present in the other three. Each of the Okinawan species has accumulated several point mutations: *Protobothrops* (Phe-73, Val-248, Ser-272, Leu-304, Thr-324, Asp-485,) and *Ovophis* (Val-73, Ile-144, Thr-176, Thr-220, Thr-396, Val-473, Glu-559, Asn-577). Click here for file

Additional file 16: Figure S9 Alignment of a partial vespryn transcript with vespryn sequences from elapid and crotalid venoms. The *Protobothrops* vespryn [AB851949] is most closely related to that from *Lachesis*, which also displays a four-residue gap from positions 25-28. Only three of the first 70 residues differ between these two toxins. The three crotalid vespryns are all 28-32 residues longer at the N-terminus than the two corresponding toxins from *Ophiophagus hannah* and *Pseudechis australis* venoms [223]. Click here for file
