# Supplementary figures and images for: Quantitative high-throughput profiling of snake venom gland transcriptomes and proteomes (Ovophis okinavensis and Protobothrops flavoviridis)
Source: BMC Genomics. 2013 Nov 14;14:790. doi: 10.1186/1471-2164-14-790 (PMC3840601; doi:10.1186/1471-2164-14-790)

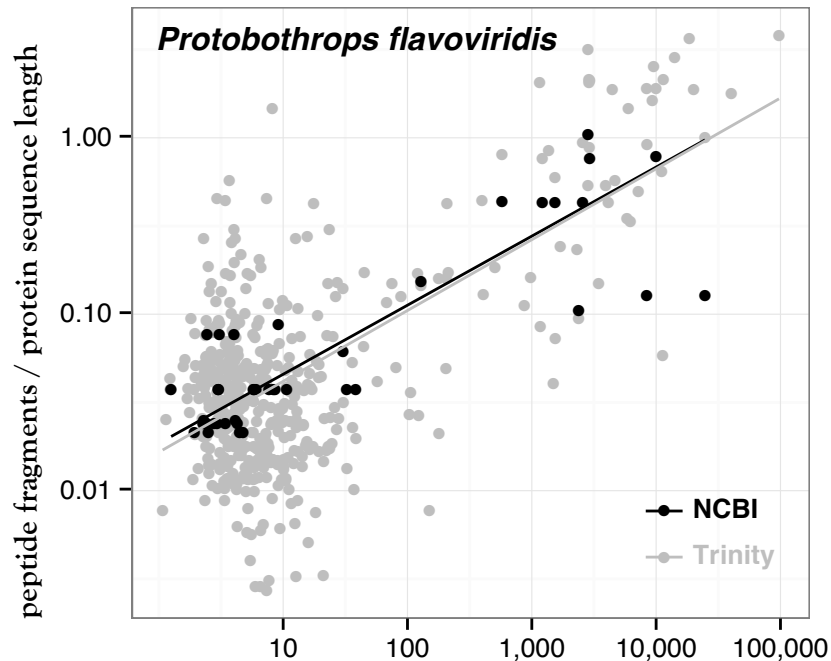

cDNA fragments / kilobase of exon / million fragments mapped (FPKM)

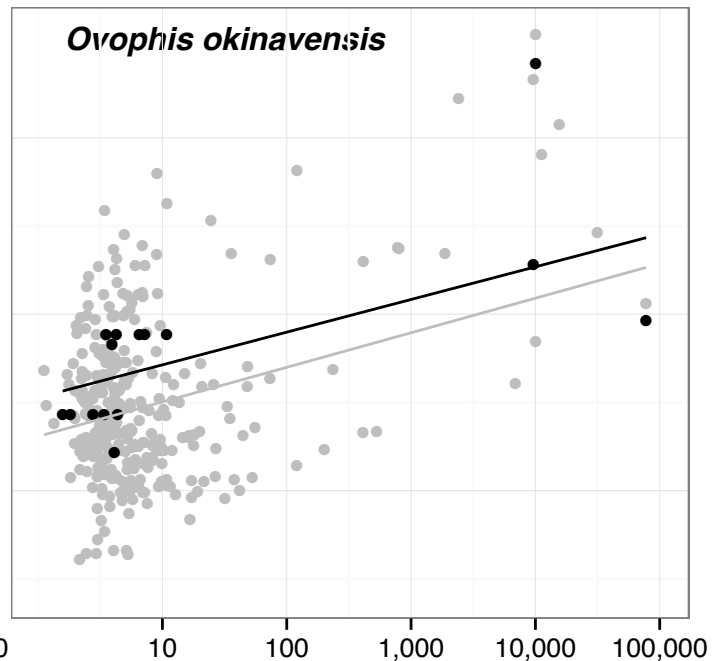

Supplement: Additional file 8: Figure S1 — Correlation between abundances of proteins predicted using NCBI data (black) and de novo assembled reference sequence (grey). Homologies between the two protein sets were determined using reciprocal best BLAST, so many of the proteins detected in the de novo transcriptome were omitted in the comparison, because they did either did not have homology to known snake proteins, or this relationship could not be determined with certainty, e.g., in the case of multiple isoforms or closely related genes. Nonetheless, the correlation coefficients were close between the two data sets, suggesting that the measure of protein abundance was robust to the choice of protein reference data set (Protobothrops: NCBI r = 0.52, p = 0.014, Trinity r = 0.64, p = 2.2e-16; Ovophis: NCBI r = 0.64, p = 1.2e-4, Trinity r = 0.68, p = 6.3e-10). Note that the correlation coefficients differ slightly with Figure 2, since the analysis presented in Additional file 8: Figure S1 did not involve assignment of unmapped proteins by PEAKS. [file 1471-2164-14-790-S8.pdf]

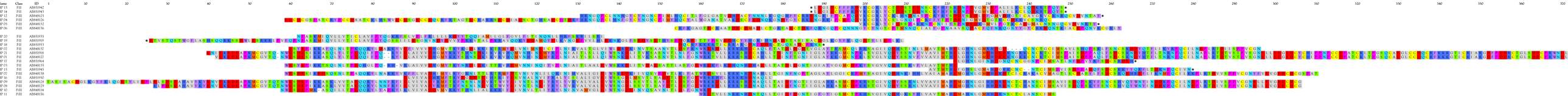

Supplement: Additional file 9: Figure S2 — Alignment of metalloproteases from the Protobothrops flavoviridis transcriptome. These sequences assort into two distinct groups, upper and lower. Members of the lower group display significant similarities and align well. Members of the upper group, for the most part, align poorly with one another, and essentially not at all with the lower group. Both groups contain both P-II and P-III MPs. Given the size of many MPs, some of these partial sequences probably represent non-overlapping segments, despite attempts by the software to align them. [file 1471-2164-14-790-S9.pdf]

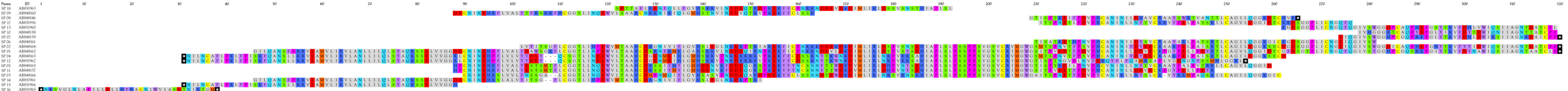

Supplement: Additional file 11: Figure S4 — Alignment of 18 serine protease sequences from the Protobothrops flavoviridis transcriptome. SP12 appears to be an inactive plasminogen activator transcript, while SP11 is probably a truncated member of the same subclass. [file 1471-2164-14-790-S11.pdf]
