# Supplementary material for: Quantitative high-throughput profiling of snake venom gland transcriptomes and proteomes (Ovophis okinavensis and Protobothrops flavoviridis)
Source: BMC Genomics. 2013 Nov 14;14:790. doi: 10.1186/1471-2164-14-790 (PMC3840601; doi:10.1186/1471-2164-14-790)
Supplement: Additional file 12: Figure S5 — Alignment of 26 Ovophis okinavensis serine protease sequences. It is impossible to infer biological activities from these transcripts; however, the Ovophis transcripts seem to fall into three or four structural subclasses or groupings. SP15 and related sequences with clusters of three acidic residues (positions 121-123) and three aromatic residues (position 132-134) appear most similar to thrombin-like enzymes. SP05 and 06 all display a high percentage of aliphatic and aromatic residues (positions 116-140), but their biological activity is not known. SP08 is apparently a thrombin-like enzyme. SP09 is most similar, based on this fragment, to an SP from Protobothrops jerdonii venom that has lost two of the three catalytic residues of active SPs. [file 1471-2164-14-790-S12.pdf]

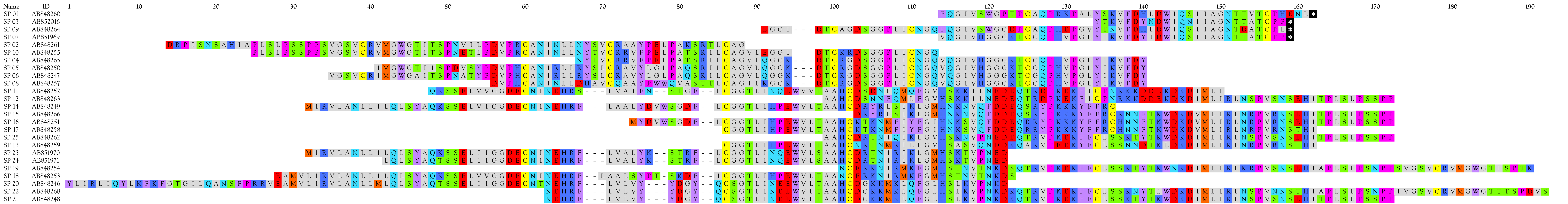

Name ID
SP 01 AB848260
SP 03 AB852016
SP 09 AB848264
SP 07 AB851969
SP 02 AB848261
SP 10 AB848255
SP 04 AB848265
SP 05 AB848250
SP 06 AB848247
SP 08 AB848257
SP 11 AB848252
SP 12 AB848263
SP 14 AB848249
SP 15 AB848266
SP 16 AB848251
SP 17 AB848258
SP 25 AB848262
SP 13 AB848259
SP 23 AB851970
SP 24 AB851971
SP 19 AB848254
SP 18 AB848253
SP 20 AB848246
SP 22 AB848268
SP 21 AB848248
