# Supplementary material for: Quantitative high-throughput profiling of snake venom gland transcriptomes and proteomes (Ovophis okinavensis and Protobothrops flavoviridis)
Source: BMC Genomics. 2013 Nov 14;14:790. doi: 10.1186/1471-2164-14-790 (PMC3840601; doi:10.1186/1471-2164-14-790)
Supplement: Additional file 13: Figure S6 — Alignment of all CTL transcripts from both venoms with sequences of convulxin (Crotalus durissus terrificus) and flavocetin (Protobothrops flavoviridis). Protobothrops venom contained various Factor IX/X binding proteins that were absent in Ovophis venom. [file 1471-2164-14-790-S13.pdf]

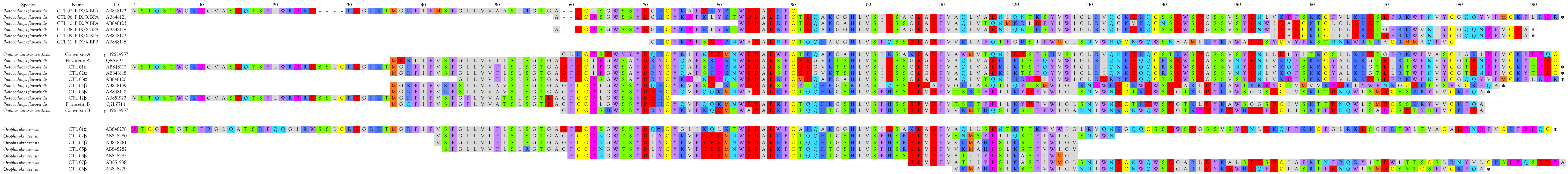

Species
Name
ID
Protobothrops flavoviridis CTL 07 F IX/X BPA AB848122
Protobothrops flavoviridis CTL 06 F IX/X BPA AB848121
Protobothrops flavoviridis CTL 10 F IX/X BPA AB848123
Protobothrops flavoviridis CTL 08 F IX/X BPA AB848119
Protobothrops flavoviridis CTL 09 F IX/X BPA AB848122
Protobothrops flavoviridis CTL 11 F IX/X BPB AB848145
Crotalus durissus terrificus Convulxin A gi 39654957
Protobothrops flavoviridis Flavocetin A Q8AV97.1
Protobothrops flavoviridis CTL 01α AB848117
Protobothrops flavoviridis CTL 02α AB848118
Protobothrops flavoviridis CTL 03α AB848120
Protobothrops flavoviridis CTL 04β AB848139
Protobothrops flavoviridis CTL 05β AB848140
Protobothrops flavoviridis CTL 12β AB851923
Protobothrops flavoviridis Flavocetin B Q7LZ71.1
Crotalus durissus terrificus Convulxin B gi 39654957
Ovophis okinavensis CTL 01α AB848278
Ovophis okinavensis CTL 02β AB848280
Ovophis okinavensis CTL 04β AB848281
Ovophis okinavensis CTL 05β AB848282
Ovophis okinavensis CTL 03β AB848283
Ovophis okinavensis CTL 07β AB851988
Ovophis okinavensis CTL 06β AB848279
