# Supplementary material for: Quantitative high-throughput profiling of snake venom gland transcriptomes and proteomes (Ovophis okinavensis and Protobothrops flavoviridis)
Source: BMC Genomics. 2013 Nov 14;14:790. doi: 10.1186/1471-2164-14-790 (PMC3840601; doi:10.1186/1471-2164-14-790)
Supplement: Additional file 14: Figure S7 — Alignment of known bradykinin-potentiating peptides from various viperid venoms showing the great sequence variability in this toxin class [78-83,191,211-222]. [file 1471-2164-14-790-S14.pdf]

Species	1				5					10				14	References
Vipera aspis	Q	G	W	P	G	P	K	V	P	P					[216]
Gloydius blomhoffii	Q	G	L	P	P	R	P	K	I	P	P				[215, 217]
Gloydius blomhoffii	Q	G	L	P	P	G	P	P	I	P	P				[215, 217]
Gloydius blomhoffii	Q	K	W	D	P	P	P	V	S	P	P				[215, 217]
Gloydius blomhoffii	Q	G	R	P	P	G	P	P	I	P					[81]
Gloydius blomhoffii	Q	G	R	P	P	G	P	P	I	P	R				[191, 217]
Gloydius blomhoffii	Q	G	L	P	P	G	P	P	I	P	R				[217]
Gloydius halys	Q	G	R	P	P	G	P	P	I	P	P				[82]
Protobothrops flavoviridis	Q	G	R	P	R	S	E	V	P	P					[78]
Protobothrops flavoviridis	Q	S	K	P	G	R	S	P	P	I	S	P			[78, present study]
Protobothrops gramineus	Q	E	K	P	G	R	S	P	P	I	S	P			[78]
Bothrops alternatus	Q	W	P	D	P	S	S	D	I	P	P				[221]
Bothrops insularis	Q	W	G	Q	H	P	N	I	P	P					[79]
Bothrops insularis	Q	N	W	P	H	P	Q	I	P	P					[79, 221]
Bothrops insularis	Q	L	G	P	P	P	R	P	Q	I	P	P			[221]
Bothrops insularis	Q	L	G	P	P	R	P	Q	I	P	P				[79]
Bothrops insularis	Q	G	G	W	P	R	P	G	P	E	I	P	P		[79, 221]
Bothrops insularis	Q	G	G	P	P	R	P	Q	I	P	P				[79]
Bothrops insularis	Q	Q	K												[79]
Bothrops insularis	Q	K	W												[79]
Bothrops insularis	Q	K	W	A	P										[79]
Bothrops jararaca	Q	K	W	A	P										[80, 83, 213, 217]
Bothrops jararaca	Q	W	P	R	P										[80]
Bothrops jararaca	Q	S	W	P	G	P									[80, 213]
Bothrops jararaca	Q	D	G	P	I	P	P								[80]
Bothrops jararaca	Q	W	P	R	P	T	P	Q	I	P	P				[80, 218]
Bothrops jararaca	Q	W	P	R	P	Q	I	P	P						[80, 213, 218]
Bothrops jararaca	Q	N	W	P	R	P	Q	I	P	P					[80, 218, 221]
Bothrops jararaca	Q	N	W	P	H	P	Q	I	P	P					[80, 217, 218]
Bothrops jararaca	Q	S	W	P	G	P	N	I	P	P					[80, 213, 217, 218]
Bothrops jararaca	Q	G	G	W	P	R	P	G	P	E	I	P	P		[80, 213, 217, 218]
Bothrops jararaca	Q	G	G	L	P	R	P	G	P	E	I	P	P		[80, 213]
Bothrops jararaca	Q	W	A	Q	W	P	R	P	T	P	Q	I	P	P	[80]
Bothrops jararaca	Q	G	R	A	P	G	P	P	I	P	P				[80, 217]
Bothrops jararaca	Q	G	R	A	P	H	P	P	I	P	P				[80, 217]
Bothrops jararaca	Q	G	W	A	W	P	R	P	Q	I	P	P			[80, 83]
Bothrops jararaca	Q	G	R	P	P	G	P	P	I	P	P				[80]
Bothrops jararaca	Q	G	G	A	G	W	P	P	I	P	P				[221]
Bothrops jararaca	Q	W	G	R	P	P	G	P	P	I	P	P			[80]
Bothrops jararaca	Q	W	A	Q	W	P	R	P	Q	I	P	P			[80]
Bothrops jararacussu	Q	G	R	P	P	G	P	P	I	P	P				[221]
Bothrops jararacussu	Q	A	R	P	P	H	P	P	I	P	P				[80,221]
Bothrops jararacussu	Q	N	W	P	H	P	Q	I	P	P					[221]
Bothrops jararacussu	Q	G	G	W	P	R	P	G	P	E	I	P	P		[221]
Bothrops neuwiedi	Q	G	G	W	P	R	P	G	P	E	I	P	P		[212, 221]
Bothrops neuwiedi	Q	W	P	R	P	T	P	Q	I	P	P				[221]
Bothrops neuwiedi	Q	W	P	R	P	Q	I	P	P						[221]
Agkistrodon piscivorus piscivorus	Q	L	W	P	R	P	H	I	P	P					[211]
Agkistrodon bilineatus			T	P	P	A	G	P	D	V	G	P	R		[222]
Lachesis muta				W	P	P	R	P	Q	I	P	P			[220]
Lachesis muta	Q	K	P	W	P	P	G	H	H	I	P	P			[220]
Lachesis muta	Q	E	W	P	P	G	H	H	I	P	P				[220]
Lachesis muta	Q	K	K	W	P	P	G	H	H	I	P	P			[220]
Lachesis muta	Q	K	W	D	P	P	P	I	S	P	P				[220]
Lachesis muta			T	P	P	A	G	P	D	V	G	P	R		[222]
Crotalus adamanteus	Q	G	G	W	P	R	N	P	I	P	P				[221]
Crotalus adamanteus	Q	Q	W	P	P	G	H	H	I	P	P				[221]
Crotalus atrox	Q	L	W	P	R	P	Q	I	P	P					[219]
Crotalus viridis viridis	Q	S	A	P	G	N	E	A	I	P	P				[221]
Crotalus viridis viridis	Q	G	G	A	P	W	N	P	I	P	P				[221]
Crotalus viridis viridis	Q	G	P	S	P	R	H	P	I	P	P				[221]
Crotalus viridis viridis	Q	N	M	E	A										Aird and Kaiser (1986, unpublished)
Crotalus viridis viridis	Q	L	W	P	R	P	H	V	P						Aird and Kaiser (1986, unpublished)
Crotalus viridis viridis	Q	G	W	P	P	G	H	H	I	P					Aird and Kaiser (1986, unpublished)
Crotalus viridis viridis			T	P	P	A	G	P	D	V	G	P	R		Aird and Kaiser (1986, unpublished), 214]
Crotalus durissus collilineatus			T	P	P	A	G	P	D	G	G	P	R		Higuchi et al. (2003, unpublished)
Crotalus durissus collilineatus	Q	R	W	P	H	L	E	I	P	P					Higuchi et al. (2003, unpublished)
Crotalus durissus collilineatus	H	R	W	P	H	P	E	I	P	P					Higuchi et al. (2003, unpublished)
Crotalus durissus collilineatus	Q	N	W	K	S	P									Higuchi et al. (2003, unpublished)
Crotalus durissus terrificus	Q	R	W	P	H	L	E	I	P	P					[221]
