# Supplementary material for: Quantitative high-throughput profiling of snake venom gland transcriptomes and proteomes (Ovophis okinavensis and Protobothrops flavoviridis)
Source: BMC Genomics. 2013 Nov 14;14:790. doi: 10.1186/1471-2164-14-790 (PMC3840601; doi:10.1186/1471-2164-14-790)
Supplement: Additional file 15: Figure S8 — Alignment of Protobothrops flavoviridis [AB851922] and Ovophis okinavensis [AB848286] dipeptidyl peptidase IV sequences with two isomers from Gloydius brevicaudus venom. The former sequences each possess a leucine residue in position 268 that is missing in the Gloydius sequences. They also have Gly-80 where Gloydius has Glu, Ile-85/Val, Asn-113/Ser, Thr-170/Ala, Ser-215/Arg, Ala-395/Ser, Arg-502/Ser, Gly-632/Asp, and Glu-680/Lys. The Protobothrops sequence lacks asparagine-133, which is present in the other three. Each of the Okinawan species has accumulated several point mutations: Protobothrops (Phe-73, Val-248, Ser-272, Leu-304, Thr-324, Asp-485,) and Ovophis (Val-73, Ile-144, Thr-176, Thr-220, Thr-396, Val-473, Glu-559, Asn-577). [file 1471-2164-14-790-S15.pdf]

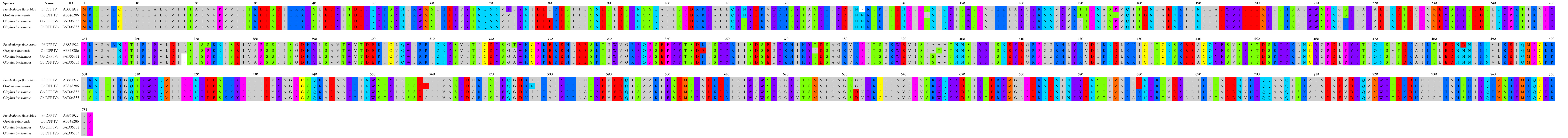

Species
Name
ID
Protobothrops flavoviridis
Pf DPP IV
AB851922
Ovophis okinavensis
Oo DPP IV
AB848286
Gloydius brevicaudus
Gb DPP IVa
BAD06332
Gloydius brevicaudus
Gb DPP IVb
BAD06333
