# Supplementary material for: Quantitative high-throughput profiling of snake venom gland transcriptomes and proteomes (Ovophis okinavensis and Protobothrops flavoviridis)
Source: BMC Genomics. 2013 Nov 14;14:790. doi: 10.1186/1471-2164-14-790 (PMC3840601; doi:10.1186/1471-2164-14-790)
Supplement: Additional file 16: Figure S9 — Alignment of a partial vespryn transcript with vespryn sequences from elapid and crotalid venoms. The Protobothrops vespryn [AB851949] is most closely related to that from Lachesis, which also displays a four-residue gap from positions 25-28. Only three of the first 70 residues differ between these two toxins. The three crotalid vespryns are all 28-32 residues longer at the N-terminus than the two corresponding toxins from Ophiophagus hannah and Pseudechis australis venoms [223]. [file 1471-2164-14-790-S16.pdf]

Species	ID	Sequence (positions 1–223)
Pseudechis australis	ABW74876.1	MLLFTLCFFADLENGGKALASPPGKWKKANVRFDSNTAFESLVVSPDKKTVENVGVPQVVPDNPERFSSSPCVLGSPGFRSGRHFFEVKYGTQREWAVGLAGKSVKRKGYLRLVPEELIWQRGLWLRRLETDSDKLQKGSGKIVVFLDYSEGKVIFDQDGEVTTVQANFNGEEVVPFYYLGGGVSLTNL
Ophiophagus hannah	P83234.2	MLLFTLCFFADQENGGKALASPPGNWQKADVTFDSNTAFESLVVSPDKKTVENVGVPKGVPDSPERFSSSPCVLGSPGFRSGKHFFEVKYGTQREWAVGLAGKSVKRKGYLRLVPEERIWQKGLWWLRRLETDSDKLQKGSGKIIVFLDYDEGKVIFDLDGEVTTIQANFNGEEVVPFYYIGARVSLANL
Crotalus adamanteus	AFJ49267.1	MSPSAGLQFSLYFLQTKKVLWKLTDKEKGLCYILLFTLCFFADQENGGKALASPPGIWKRADVTFDSNTAFSSLVVSANKKTVKNVGVPQVVPDNPERFNSSPCVLGSPGFRSGKHYFEVKYGTQREWAVGIAGKSVKRKGNLMLVPEERIWQMGLWWLRHLETDPGRVHSTSGKITVFVDYNGGNVIFDLNRINTTLKANFNGEEVVPFFYLGGTVSLTTL
Lachesis muta	Q27J48.1	MSPSAGFQFSLYFLQTKKVLWKLT----GLCYILLFTLCFFADQENGGKALASPPGIWKRADVTFDSNTAFSSLVVSANKKTVKNVGVPQVVPDNPERFNSSPCVLGSPGFRSGKHYFEVKYGTQREWAVGLAGKSVKRKGNLNLVPEERIWQTGLWWLRHLETDPGRVHSTSGKITVFVDYSGGNVIFDLNRIITILKANFSGEEVVPFFYLGGTVSLTTL
Protobothrops flavoviridis	AB851949	MSPSAGFQFSLNFLQTKKVLWKLT----GLCYILLFTLCFFADQKNGGKALVLPPGIWKKADVTFDSNTAFSSL
