# Supplementary material for: Quantitative high-throughput profiling of snake venom gland transcriptomes and proteomes (Ovophis okinavensis and Protobothrops flavoviridis)
Source: BMC Genomics. 2013 Nov 14;14:790. doi: 10.1186/1471-2164-14-790 (PMC3840601; doi:10.1186/1471-2164-14-790)
Supplement: Additional file 10: Figure S3 — Alignment of metalloproteases from the Ovophis okinavensis transcriptome, showing the sequences of five P-II and three P-III MPs. [file 1471-2164-14-790-S10.pdf]

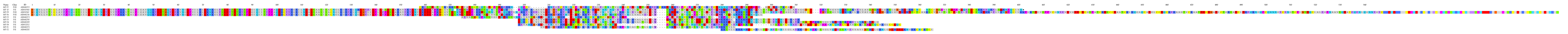
Name
Class
ID
1
10
MP 07
P-III
AB848272
MP 10
P-II
AB851968
MP 09
P-III
AB851967
MP 06
P-III
AB848256
MP 03
P-II
AB848273
MP 05
P-II
AB848290
MP 04
P-II
AB848289
MP 08
P-II
AB852012
MP 01
P-II
AB848275
MP 02
P-II
AB848287
